# An AP-MS- and BioID-compatible MAC-tag enables comprehensive mapping of protein interactions and subcellular localizations

**DOI:** 10.1038/s41467-018-03523-2

**Published:** 2018-03-22

**Authors:** Xiaonan Liu, Kari Salokas, Fitsum Tamene, Yaming Jiu, Rigbe G. Weldatsadik, Tiina Öhman, Markku Varjosalo

**Affiliations:** 1 Institute of Biotechnology, University of Helsinki, Helsinki, 00014 Finland; 2 Helsinki Institute of Life Science, University of Helsinki, Helsinki, 00014 Finland; 3 Proteomics Unit, University of Helsinki, Helsinki, 00014 Finland

## Abstract

Protein-protein interactions govern almost all cellular functions. These complex networks of stable and transient associations can be mapped by affinity purification mass spectrometry (AP-MS) and complementary proximity-based labeling methods such as BioID. To exploit the advantages of both strategies, we here design and optimize an integrated approach combining AP-MS and BioID in a single construct, which we term MAC-tag. We systematically apply the MAC-tag approach to 18 subcellular and 3 sub-organelle localization markers, generating a molecular context database, which can be used to define a protein’s molecular location. In addition, we show that combining the AP-MS and BioID results makes it possible to obtain interaction distances within a protein complex. Taken together, our integrated strategy enables the comprehensive mapping of the physical and functional interactions of proteins, defining their molecular context and improving our understanding of the cellular interactome.

## Abstract (web summary)

AP-MS and BioID provide complementary insights into cellular protein interaction networks. To facilitate their combined use, the authors here present an AP-MS- and BioID-compatible affinity tag, enabling efficient determination of cellular protein locations and interaction distances.

## Introduction

Majority of proteins do not function in isolation and their interactions with other proteins define their cellular functions. Therefore, detailed understanding of protein–protein interactions (PPIs) is the key for deciphering regulation of cellular networks and pathways. During the last decade, the versatile combination of affinity purification and mass spectrometry (AP-MS) revolutionized the detailed characterization of protein complexes and protein-interaction networks^1^. The AP-MS approach relies on expression of a bait protein coupled with an epitope tag or antibodies targeting the endogenous bait protein, allowing purification of the bait protein together with the associating proteins (preys). This approach has been proven well suited for even large-scale high-throughput studies, and to yield highly reproducible data in both intra- and inter-laboratory usage^2^. The most commonly used epitope tags in medium to large-scale studies include FLAG^3^, His^4^, MYC^5^, HA^6^, GFP^7^ and Strep^8^, of which the Strep-tag has become the gold-standard in affinity purification proteomics due to unparalleled protein purity in physiological purification conditions as well as the possibility for native competitive elution using biotin.

AP-MS can also be combined with quantitative proteomics approaches to better understand the protein complex stoichiometry^9^ and the dynamics of protein–complex (dis)assembly^1,10^. The combination of AP-MS with other techniques, such as biochemical fractionation, intact mass measurement and chemical crosslinking^11,12^, has been used to characterize supramolecular organization of protein complexes.

Although AP-MS remains the most used method for mapping protein-protein interactions, the recently developed proximity labeling approaches, such as BioID^13^ and APEX^14^, have become complementary and somewhat competing methods. BioID involves expression of the protein of interest fused with a prokaryotic biotin ligase (BirA) and the subsequent biotinylation of the amine groups of the neighboring proteins when excess of biotin is added to the cells. Whereas the wild-type BirA from *E. Coli* is capable of transferring the biotin only to a substrate bearing a specific recognition sequence, the generation of a promiscuous BirA* (Arg118Gly mutant) allows the biotinylation of any protein found within a 10 nm labeling radius^13,15^. While BioID has the abilities to capture weak and/or transient protein-protein interactions, the identified interactions are not limited to direct binders but can include proximate proteins as well.

In order to avoid artefactual interactions caused by overexpression of the bait proteins, majority of the large-scale interaction proteomic studies employ the Flp-In T-REx 293 cell line allowing moderate and inducible bait protein expression from isogenic cell clones^16^. Although the system allows rapid generation of transgene stably expressing cell lines, comprehensive analyses utilizing complementarily both AP-MS and BioID is resource-intense in the respect of construct and cell line generation. To address this caveat and allow high-throughput comprehensive interactome analyses, we generated a Gateway®-compatible MAC (Multiple Approaches Combined) -tag enabling both the single-step Strep AP-MS as well as the BioID analysis with a single construct and with single affinity reagent, which decreases the number of required individual cell lines by 50% and should improve the data reproducibility, respectively. In addition to allow visualization of tagged bait protein by immunohistochemistry, we included as well a nine amino acid hemagglutinin (HA)-epitope. The HA epitope also facilitates additional follow-up approaches such as ChIP-Seq^17^ and purification of the crosslinked proteins for cross-linking coupled with mass spectrometry (XL-MS)^18^, making the MAC-tag almost as versatile as the Swiss Army knife.

To benchmark the usability and performance of the MAC-tag we applied it to 18 bona fide subcellular localization marker proteins. This allows us to validate the correct localization of the MAC-tagged marker proteins as well as to monitor the localization of the in vivo biotinylated interactors. These interactions also provide information about the cellular functions of the 18 marker proteins. Furthermore, the 18 localization markers and their 1911 interactions form a reference molecular context repository, which can be used for ‘mass spectrometry (MS) microscopy’ analysis of a protein of interest. Moreover, the combined analysis with AP-MS and BioID allows deriving relative spatial distances for proteins within a complex. Taken together, the MAC-tag and corresponding analysis approaches provide a plethora of information on the cellular functions and the molecular context of proteins.

## Results

### Development of MAC-tag based AP-MS and BioID pipelines

**Fig. 1:**
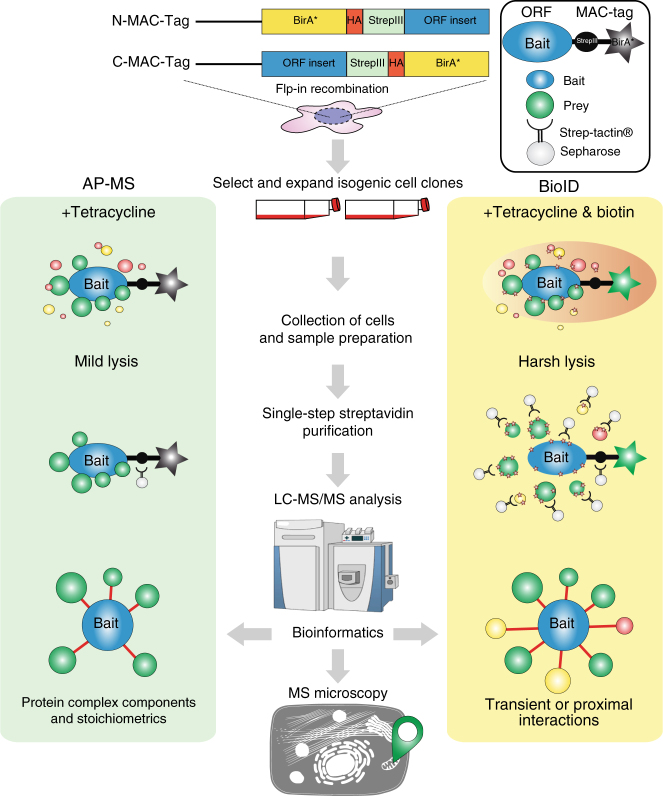
MAC-tag-based workflow for identification of protein complexes and interactions. Gateway compatible MAC-tag destination vectors containing StrepIII, HA and BirA* were designed to allow the gene of interest either C-terminal or N-terminal tagging. The expression vector can then be transfected into Flp-In T-REx 293 to establish the transgenic stably and inducible expressing isogenic cell lines. For the AP-MS and BioID analysis approaches, the cell line is separated into two cultures, BioID cells receiving addition of 50 μM biotin in their culture medium. In the following protein extraction process, optimized lysis and affinity purification conditions for both analysis approaches were used. The interacting proteins were then analyzed by quantitative mass spectrometry and high-confidence interaction proteins (HCIPs) were inferred via stringent statistical filtering. This integrated workflow allows laborless generation of cellular material for analyses, and results in integrated view of the formed protein complexes, protein-protein interactions and detailed molecular context definition

To generate a versatile approach for identification of both stable physical and transient functional protein-protein interactions we integrated and optimized the BioID approach with our single-step Strep AP-MS pipeline^10,19^. Both of these approaches have become the method of choice for interactomics analyses. We have recently shown the effectiveness of using these approached complimentarily^10,19^. However, the complementary use of the two techniques has been labor-intense, involving tagging of the bait proteins with BirA* and Strep-tag individually, as well as generation of two set of cell lines per bait. To overcome the major limitations, we have developed an integrated experimental workflow utilizing a MAC-tag containing both StrepIII-tag and BirA* (Supplementary Fig. 1a). In addition to optimizing the experimental steps, we focused on the compatibility of the two methods and to the simplicity of the analysis pipelines to generate a process with improved performance and reproducibility on detecting protein-protein interactions. In contrast to coupling BirA* with epitope tags such as Myc^10^ or FLAG^20^, the two MAC-tag pipelines differ only in the activation of the BirA* by addition of biotin to the cell culture media and harsher lysis condition in the BioID pipeline (Fig. 1 and Supplementary Fig. 1a, b). Without biotin addition the BirA* in the MAC-tag is inactive (Supplementary Fig. 1b, c), resulting in identical (cor = 0.88–0.99) single-step affinity purification results as vector with only StrepIII-tag (Supplementary Fig. 1d, e). Similarly, when biotin was added the results compare (cor = 0.95–0.97) to that of a vector with BirA* (Supplementary Fig. 1d, e and Supplementary Data 1d).

The developed integrated approach significantly enhances (> two-fold) the throughput of generating bait-expressing cell lines, facilitates a comprehensive analysis of protein-protein interactions utilizing both the BioID and AP-MS, and allows analysis of protein complexes and even transient functional interaction networks with high sensitivity and reproducibility. Additionally, the MAC-tag allows visualization of the bait protein with anti-HA antibody detecting the HA epitope. This versatility of our approach was expected to give detailed view on the bait protein formed complexes, interactions, and actual molecular context via the detected stable, transient and/or proximal interactions.

### MAC-tagged cellular localization markers localize correctly

**Fig. 2:**
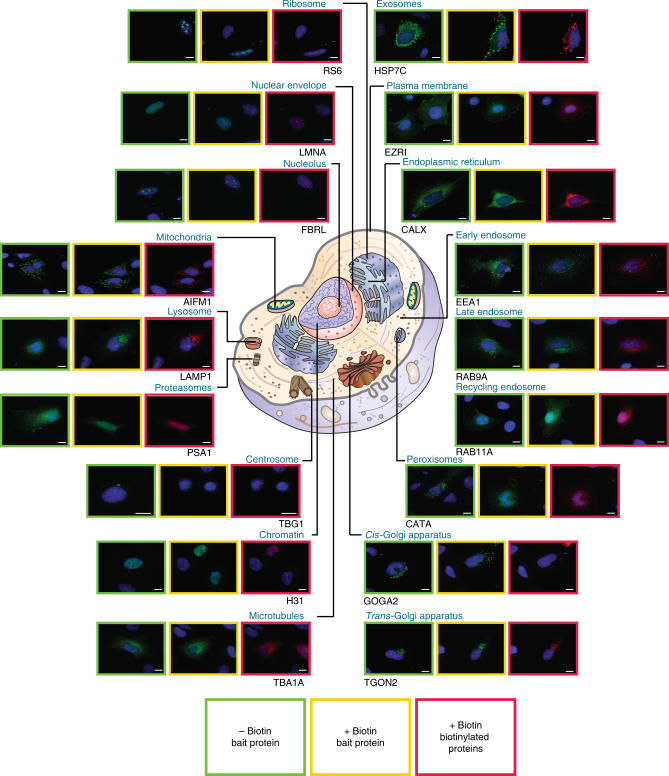
Fluorescence microscopy analysis of the cellular localization markers. The 18 subcellular localization markers fused with MAC-tag were visualized by immunofluorescence staining using Alexa Fluor 488 labeled anti-HA immunostaining (green), their in vivo biotinylated interactors with Alexa Fluor 594 streptavidin (red), and cell nuclei with DAPI (blue) (Scale bar: 10 μm)

We then went on and evaluated the MAC-tag system with 18 bona fide cellular localization markers (Supplementary Data 1a) that cover most of cellular organelles to have more comprehensive view of the application of our integrated multiple approach system. Initially 18 localization markers were cloned to the MAC-tag vector, and as a first step we explored their localization using fluorescence microscopy. The tagged-localization markers were visualized with anti-HA antibody and the in vivo biotinylated interactors with Alexa Fluor 594 streptavidin (Fig. 2). These subcellular markers included: mitochondria (Apoptosis-inducing factor 1, AIFM1); endoplasmic reticulum (Calnexin, CALX); peroxisome (Catalase, CATA); early endosome (Early endosome antigen 1, EEA1); cytoplasmic peripheral plasma membrane marker (Ezrin, EZRI); nucleolus marker (rRNA 2′-O-methyltransferase fibrillarin, FBRL); *cis*-Golgi marker (Golgin subfamily A member 2, GOGA2); chromatin (Histone H3.1, H31); exosome (Heat shock cognate 71 kDa protein, HSP7C); lysosome (Lysosome-associated membrane glycoprotein 1, LAMP1); nuclear envelope marker (Prelamin-A/C, LMNA); proteasome (Proteasome subunit alpha type-1, PSA1); recycling endosome (Ras-related protein Rab-11A, RAB11A); late endosome (Ras-related protein Rab-9A, RAB9A); microtubule (Tubulin alpha-1A chain, TBA1A); centrosome (Tubulin gamma-1 chain, TBG1); *trans*-Golgi (*trans*-Golgi network integral membrane protein 2, TGON2); and ribosome (40S ribosomal protein S6, RS6) (Supplementary Data 1a). All of the 18 MAC-tagged marker proteins localized to their corresponding well documented cellular compartments, illustrating that the MAC-tag or the activation of the BirA* does not change the correct localization of these proteins. We also verified this for several of the used marker proteins (11/11 tested) using specific antibodies against the corresponding endogenous protein (Supplementary Fig. 2 and Supplementary Data 1a). Furthermore, the localization of the in vivo biotinylated interactors correlates well with that of the corresponding localization marker (Fig. 2). In addition to verifying the correct localization of marker proteins, the results highlight the usability of our MAC-tag constructs for fluorescence microscopy on detecting both the tagged protein of interest as well as the interacting proteins.

### Identifying the interactions of the localization markers

**Fig. 3:**
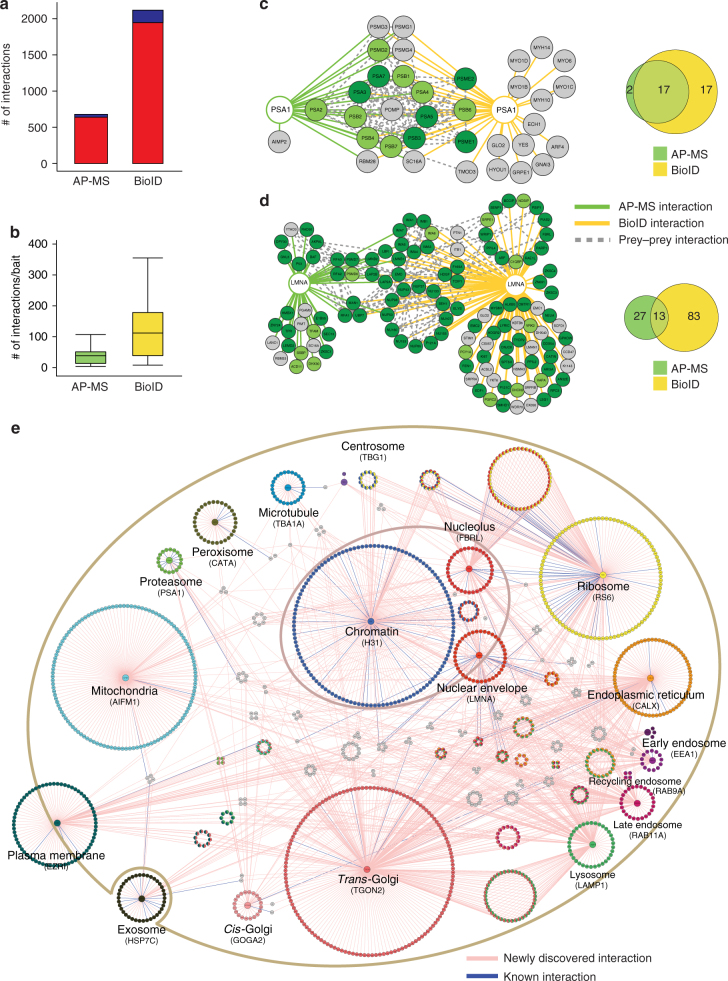
Generation of comprehensive interactome maps for the bona fide cellular markers. The 18 localization markers we subjected to our integrated analysis, resulting in identification of 679 HCIs from the AP-MS and 2118 HCIs from BioID analysis. **a** The distribution of the number of known (blue) and newly identified (red) interactions within 18 bona fide subcellular organelle/structure markers, illustrate the need for systematic analyses. **b** The distribution of the number of interactions per localization marker by AP-MS or BioID purification approach shows similar distribution of connectivity as other publications using these approaches individually. Boxplots show the median, the 25th and 75th percentile, Tukey whiskers (median ± 1.5IQR). **c**, **d** The protein-protein interaction network and molecular context for proteasome organelle marker (PSA1) and nuclear envelope (LMNA). The HCIs that were identified from AP-MS (green line) and BioID (yellow line) are shown together with the known prey-prey interactions (dashed gray line). The nodes are color-coded based on the localization rank obtained from the CellWhere database (key: dark green = primary cellular localization for the corresponding protein, light green = possible localization, gray = different or no localization assigned for the protein). The Venn diagram highlights the complementary nature of the AP-MS and BioID approaches. **e** The reference molecular context map for the 18 subcellular organelles/structures. The unique high-confidence interactors from the BioID analysis are arranged in a circle around the corresponding localization marker and the shared interactors are shown with corresponding colors representing multiple localizations. Preys with more than four subcellular localizations are shown in gray color. The newly identified interactions are shown in pink edges and the known interactions with blue

Although many proteins and proteins families have been extensively studied with wide-range of cell biological or biochemical methods, others and we have shown the AP-MS and BioID can reveal wealth of new molecular and functional information^10,20,21^. However, for many proteins not much is known and there has not been systematic methods to efficiently and comprehensively characterize them. As shown in Fig. 2, the resolution of standard fluorescence microscopy does not allow capturing information of the protein dynamic localization and molecular context. Therefore, we MAC-tagged 18 known cellular localization markers and subjected them to our integrated method to obtain detailed molecular context proteome map with information from both the physical and functional interactions formed by these proteins. The analysis resulted in 26527 interactions from BioID and 9390 from AP-MS, of which 2118 high-confidence interactions (HCIs) from BioID and 679 interactions from AP-MS were retained after using stringent statistical filtering (Fig. 3a, b and Supplementary Data 1c). The identified average connectivity (38) of the 18 localization markers, identified using AP-MS, matches well with the published large-scale studies^10,22^. As the BioID is also able to capture highly transient and close-proximity interactions, the total number of identified interactors as well as interactions per bait is higher than that of AP-MS (Fig. 3a, b). This is seen for example with Rab9A and Rab11A, two regulators of endosomal transport, for which BioID provides 16 times and 11 times more high-confidence interacting proteins (HCIPs) than AP-MS, respectively (Supplementary Fig. 3). In this case, the proteins detected solely with BioID likely represent cargo proteins in endosomal transit. Interestingly the ratio of newly identified vs. known interactions in total is almost two-fold higher with BioID (11.3) than with AP-MS (6.8), potentially reflecting the sensitivity of BioID to identify more transient and proximal interactions (Fig. 3a).

However, the complementary nature of these two methods is illustrated by their overlap as well as with their individually detected interactions, such as the ones formed by proteosomal marker PSA1^23^ and nuclear envelope marker LMNA (Fig. 3c, d). With PSA1 the overlap of AP-MS (green edges) and BioID (yellow edges) identified interactions is 17 components of the 20S core proteasome complex involved in the proteolytic degradation of most intracellular proteins (Fig. 3c and Supplementary Data 1c). BioID also captures myosins (MYH10 and MYH14) and unconventional myosins (MYO1B-D and -6), which have high turnover rates and after use they are either refolded for reuse or degraded by the proteasome^24^. Additionally, BioID identifies proteasome activator complex subunits 1 (PSME1) and 2 (PSME2), which are part of the 11S (PA28) immunoproteasome^25^.

LMNA is a component of the nuclear lamina, playing an important role in nuclear assembly, chromatin organization, and framework for the nuclear envelope and telomere dynamics. Not surprisingly both the AP-MS and BioID identify interactions with lamin-B (LMNB)1, LMNB2, lamin-B receptor (LBR), lamina-associated polypeptide (LAP)2A and LAP2B, inner nuclear membrane protein Man1 (MAN1), and emerin (EMD). Another group of interacting proteins are nuclear pore complexes (NPCs) components: nuclear envelope pore membrane protein POM (P121A, P121C), Nuclear pore complex protein Nup (NU) 107, NU133, NU153, NU155, NU160, NUP50, NUP85, NUP98, NUP37, NUP43, nucleoporin SEH1, nucleoprotein TPR, ELYS and SEC13, of which only components of the nuclear basket NUP50, TPR, SEC13 are detected of low abundance with AP-MS (Fig. 3d, and Supplementary Data 1c). This suggests that for the correct localization to the nucleoplasmic side of the nuclear envelope, LMNA needs to pass through nuclear pore and during this process it transiently interacts and in vivo biotinylates the NPCs components. Similarly, the importin transport proteins importin subunit alpha (IMA1, IMA3, IMA4, IMA5, IMA6 and IMA7), importin subunit beta-1(IMB1) are detected with BioID, but only IMA5 and IMA7 in AP-MS. In addition, several histone modifiers and chromatin remodelers are detected (AN32E, EDF1, MYSM1 and PARP1). Somewhat surprisingly our analysis also identifies several proteins involved in cell cycle and mitosis (RAD50, ARF, KI67, HDGR2, WRIP1, AKP8L, BCCIP and P53).

Both of the examples show that the detected high-confidence interacting proteins are highly specific for the studied location, as illustrated by the retrieval of the HCIPs localization information from CellWhere database^26^ (Fig. 3c, d). The proteins with the highest ranking for the particular location from CellWhere are shown in dark green and for the rest of the ranks the node color is light green. Proteins with no CellWhere ranking are shown in gray (Fig. 3c,d and Supplementary Fig. 3).

### Molecular context map reveals organelle-specific profiles

In addition to lacking molecular level resolution, standard fluorescence microscopy is often used to produce static images representing the particular time point when the image is taken. However, cellular proteins are highly diverse in their spatiotemporal properties, thus making their characterization with microscopy alone extremely challenging. The BioID, in principle, overcomes these limitations as monitoring of the biotinylated close-proximity proteins and their quantities should allow defining the BirA* -tagged bait proteins detailed molecular context within certain time period (Supplementary Fig. 3). Using the 2118 high-confidence interactions from BioID, we generated a cellular compartment-specific protein interaction map to the 18 bona fide localization markers (Fig. 3e and Supplementary Data 1c). The HCIPs domains as well as the gene ontology (GO) term profiles for each marker were unique (Supplementary Data 2a-f and Supplementary Figs 4, 5). However, we identified also shared HCIs between the endomembrane system consisting of ER (CALX), the Golgi (GOGA2 and TGON2), endosomes (EEA1, RAB9A and RAB11A) and lysosome (LAMP1). The four organelles shared 17 interactors, and the combination of any three locations shared in total 87 interactions (Supplementary Fig. 6a). These four organelles are involving in two major intracellular trafficking pathways: The exocytic pathway (ER via Golgi (53 shared interactions) to the plasma membrane); and the endocytic pathway (plasma membrane via endosomes to (1) Golgi (101 interactions) and (2) lysosome (61 interaction) to ER (86 interactions)). This organization is also well visible with, within a cell, the physically farthest from each other locating endosome and ER, sharing the least interactions of the all possible binary combinations of the four locations.

**Fig. 4:**
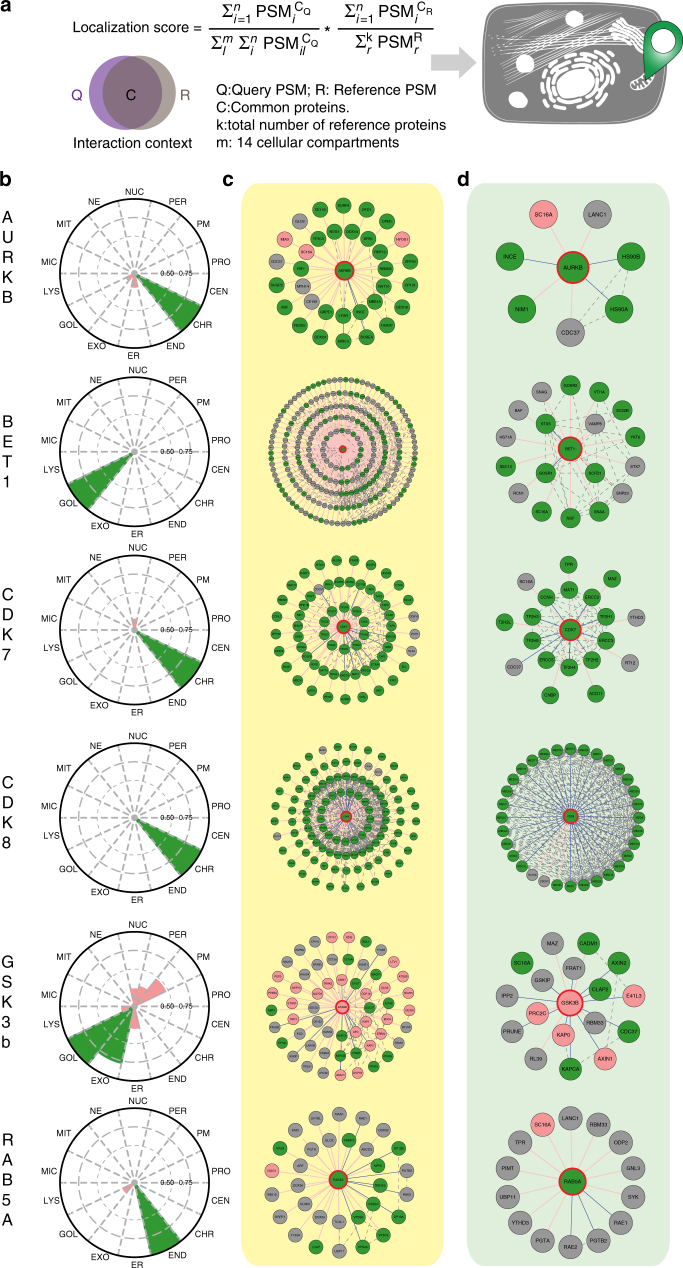
Molecular level localization mapping using the reference molecular context maps. **a** The schematic overview of the MS microscopy approach to assess the queried protein localization using our reference interaction context. **b** The polar plot shows the location of query protein observed by MS microscopy. Each sector represents one subcellular location defined by our reference database. The color assigned to each of the localization is based on the annotation frequency (Pink: 0–0.5; Yellow: 0.5–0.75; Green: 0.75–1). **c**, **d** The PPI network obtained from BioID and AP-MS are shown separately. The localization of prey proteins was verified by CellWhere database. Node color scheme coordinates the observation localization from Fig. 4b

Similarly, chromatin, nucleolus and nuclear envelope are all sub-structures in the nucleus and shared interactors with each other (Supplementary Fig. 6b); nuclear envelope (LMNA) 22 with chromatin (H31) and chromatin 31 with nucleolus (FBRL). We previously already discussed the role of nuclear envelope on chromatin organization, and chromatin control the structure of nucleolus via ribosome DNA. Nucleolus is the place where ribosomal RNA transcription and the ribosome assembly occur. Ribosome (RS6) was detected as an outlier as it shared many of the interactors with other localization markers. This is explained by the fact that protein translation requires ribosomes, which are after the synthesis of the MAC-tagged protein immediately in vivo biotinylated in the BioID approach. Therefore the ribosome (RS6) was excluded from the further analyses. However, the other localization markers, such as mitochondria (AIFM1), cytoplasmic peripheral plasma membrane (EZRI), exosome (HSP7C), peroxisome (CATA), microtubule (TBA1A), proteasome (PSA1) and centrosome (TBG1) had highly unique molecular context signature, which suggest the usability of this reference set in tracking of protein of interests dynamic localization in intracellular environment. Additionally comparison of the HCIPs cellular locations from CellWhere database showed them to be assigned to the correct cellular localization according to their bait protein, further reinforcing the idea that proteins that share their interaction profiles are proximal. Finally, total of 1911 HCIs (excluding RS6 interactions), collapsed to 14 subcellular localizations were integrated to build up the reference molecular context map (Fig. 3e). Overlaying of the protein of interests BioID PPIs with our molecular map should allow defining the dynamic localization of the protein. In principle, the developed MS microscopy approach could have high impact on cellular quantitative biology (Fig. 4a).

### MS microscopy using the molecular context map as a reference

Despite the biological significance of dynamic subcellular localizations of proteins, simple tools for detecting the relative subcellular distribution have not been extensively developed. To test the applicability of the MS microscopy on this, we selected dynamic cytoplasmic signaling molecules aurora kinase B (AURKB), cyclin-dependent kinase (CDK) 7, CDK8, and glycogen synthase kinase-3 beta (GSK3B), as well as additional markers for cellular locations ras-related protein Rab-5A (RAB5A) and Golgi vesicular membrane-trafficking protein p18 (BET1) and applied our approach to them (Fig. 4b–d, Supplementary Data 1a–c, and Supplementary Fig. 7). Aurora kinase plays an important role in cellular division by controlling chromatic segregation, which matches well to its interactions overlaying with chromatin marker H31 (Fig. 4b–d). Similarly SNARE protein BET1, involved in the docking process of ER-derived vesicles with the *cis*-Golgi membrane is assigned to Golgi (Fig. 4b–d). Essential component of the transcription factor II H (TFIIH), CDK7, and mediator complex associating CDK8 are predominantly associating with chromatin (Fig. 4b–d). This finding is in line with their important role in transcription regulation. Importantly, these examples show the high resolution of the MS microscopy to distinct exact molecular locations, which could not be resolved by fluorescence microscopy (Supplementary Fig. 7). Glycogen synthase kinase β (GSK3B) phosphorylates many substrates in mammalian cells, and functions in many physiological processes, and acts as an important regulator in Wnt and Hedgehog signaling pathways^27^. Somewhat, to our surprise our MS microscopy showed GSK3B localization to Golgi and exosomes. Recent research have demonstrated that a portion of GSK3B is localized to the *trans*-Golgi network through peripheral protein p230^28^ and that cytoplasmic GSK3B relocalizes to the same endosome as the internalized Wnt ligand^29^. It is plausible that this colocalization of GSK3B continues with active Wnt through endosomal organelles onto exosomes^30^. For validation of our endosomal location, we choose RAB5A which is known to localize to early endosomes and is involved in the recruitment of RAB7A and the maturation of these organelles to late endosomes^31^. In our analysis we can confirm the (early)-endosomal location as well as detect a fraction of Golgi localization, which could be related to the fusion of *trans*-Golgi network-derived vesicles with the early endosome^32^.

Our generated reference molecular context proteome map could be expected to be applicable with previously published heterogenous BioID datasets and even with other cell types. To test this we selected seven studies^13,21,33–37^ using BioID with heterogeneous protocols and processed the reported interactions against our reference molecular context proteome map. With this analysis we could show that our approach identified the correct subcellular localization for 22/25 of the tested experiments (Supplementary Data 3, http://www.biocenter.helsinki.fi/bi/protein/msmic/example.pdf), validating and further extending the general usability of our MS microscopy approach. Additionally, we selected two commonly used human cell lines, the human bone osteosarcoma U-2 OS and the human prostate cancer DU-145 and generated cell line specific reference molecular context proteome maps. The 17 used localization markers in both cell lines align well with their respective localization, when processed against the Flp-In  T-REx 293 reference molecular context proteome map (Supplementary Fig. 8, 9 and Supplementary Data 1c). We then compared the BioID results of AURKB, BET1, CDK7, CDK8, GSK3B and RAB5A from the Flp-In T-REx 293 (Fig. 4c and Supplementary Data 1c), with the U-2 OS, DU-145 and Flp-In T-REx 293 generated maps, and the results align extremely well (Supplementary Fig. 10).

These examples clearly establish the applicability of the MS microscopy in defining the molecular context of many protein(s), and also it´s expandability to cover also different cell types or sub-organelle structures. In addition to analysis of wild-type proteins our system should be useful for defining possible altered molecular context in human diseases caused by either somatic or germline genetic alteration, as well as for example analyzing functions of transgenic proteins not expressed in human cells.

### Molecular localization of the transgenic alternative oxidase

**Fig. 5:**
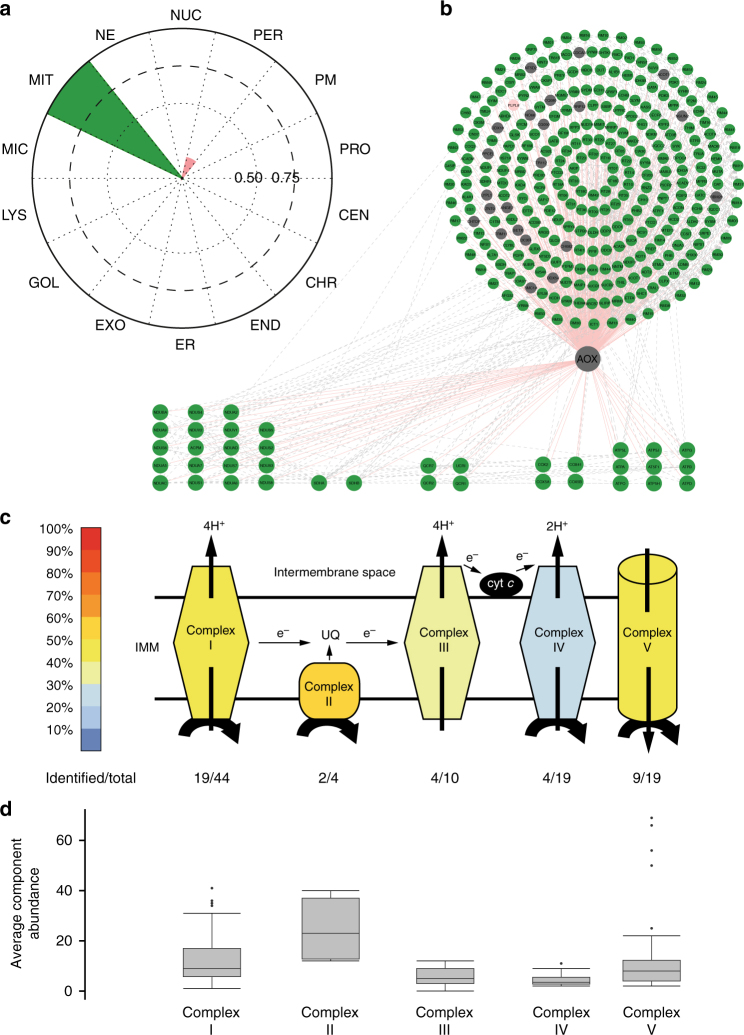
AOX localizes to inner mitochondrial membrane and in the vicinity of complex II. **a** The AOX from *Ciona Intestinalis*, was introduced to human cells and the MS microscopy assigns the AOX to localize to mitochondria. **b** The BioID approach identifies 333 interactions of which 93.1% (310) were mitochondrial (green), 0.3% (1) was peroxisomal (pink) and 6.6% (22) were unassigned (gray), based on CellWhere database. **c** Total of 38 of the interactors were components of the mitochondrial respiratory chain complexes (complex I- V, key: color gradient indicates the percentage of proteins of the each individual complexes identified). **d** The average component abundance shows that the AOX associates most with the complex II, which is in agreement with the AOX suggested functional role. Boxplots show the median, the 25^th^ and 75^th^ percentile, Tukey whiskers (median ± 1.5IQR) and outliers (.)

Alternative oxidase (AOX) present in many lower eukaryotes, but not in vertebrates, transfer electrons directly from ubiquinol to oxygen in a non-proton-motive manner^38^. Transgenic expression of AOX in mammalian systems has been suggested as a therapeutic option for treating mitochondrial disease induced by OXPHOS dysfunction^39^, and additionally it has been shown that even broad expression of AOX does not disturb normal physiology in mice^40^. The exact molecular context of AOX in mitochondria membrane is not currently known, but it is thought to locate close to complex II based on its alleviating effects after toxic or pathological inhibition of the mitochondrial respiratory chain^41^. Therefore, we decided to apply our MS microscopy method to define the molecular context of *Ciona intestinalis*’ AOX in human cells and possibly shed some light on its interactions with respiratory chain. Our approach identifies AOX predominantly to localize to mitochondria (Fig. 5a and Supplementary Fig. 7). More specifically, >90% of AOX interactors (Fig. 5b) belong to mitochondria according to CellWhere database and 48% among them have the GO term annotation of mitochondria inner membrane. Furthermore, 38 of the interactors are components of the mitochondrial respiratory complexes I–V. From these AOX prefers interactions with complex II (2/4 components detected), complex I (19/44) and complex V (9/19) (Fig. 5c), which is also visible from the quantitative interactor abundance (Fig. 5d). The detected higher quantitative abundance of the complex II proteins is in agreement with the immunoelectron microscopy findings of the complex II locating on the inner mitochondrial membrane^42^, whereas the other complexes (I,III,IV and V) would be on the cristae membrane. Therefore, our MS microscopy findings confirm the functionally suggested location (IMM, in vicinity of complex II) of transgenic AOX in human cells.

### Sub-organelle level resolution of the MS microscopy

**Fig. 6:**
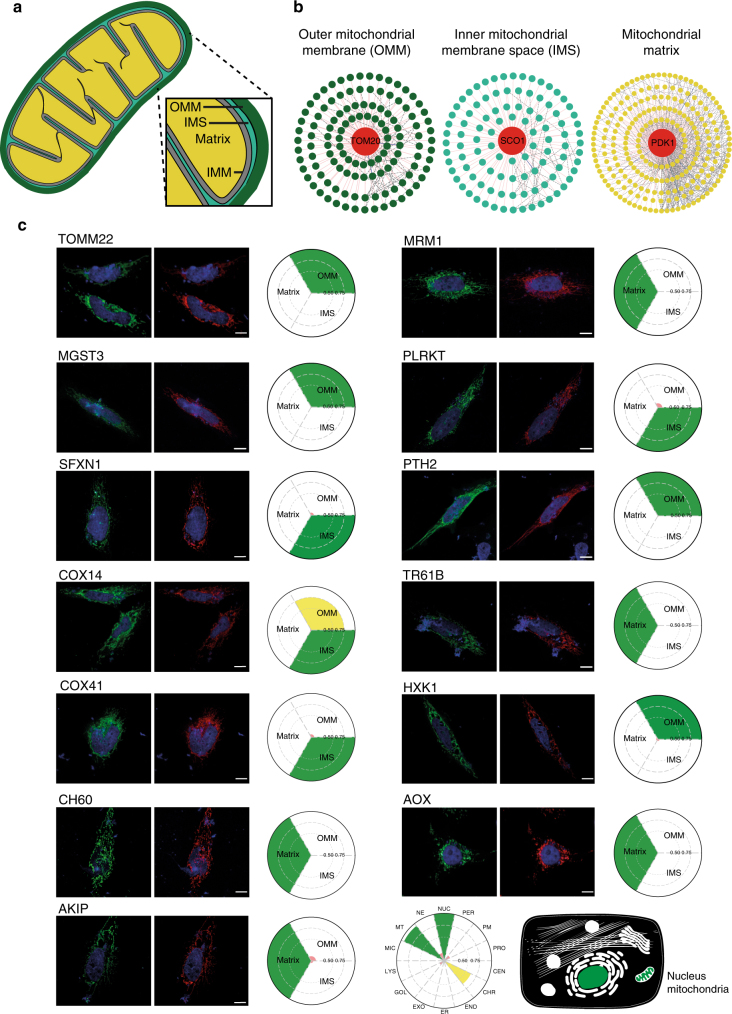
Sub-organelle level molecular context map of the mitochondria. **a** Mitochondrion can be divided to four compartments, namely to the outer mitochondrial membrane (dark green), inner mitochondrial space (light green), inner mitochondrial membrane (gray) and the mitochondrial matrix (yellow). **b** The three mitochondrial proteins, TOM20, SCO1 and PDK1, used for generation of the mitochondrial sub-organelle molecular context map, with their PPI network obtained from the BioID (key: the interacting proteins are colored according to their corresponding bait mitochondrial location. Known (blue), newly identified (red) and prey-prey (black dashed line) interactions are color-coded). **c** Confocal microscopy analysis fails to provide sub-organelle level information of mitochondrial protein, whereas MS microscopy allows assigning the proteins within mitochondrial compartments. Confocal microscopy (HC PL APO 93×/1.30 GLYC motCORR) was applied to observe the mitochondrial localization MAC-tagged mitochondrial proteins. The MAC-tagged bait proteins are visualized with anti-HA immunostaining (green), nucleus with DAPI (blue), and mitochondria by co-transfection with pDsRed-Mito vector (red), Scale bar: 10 μm. The MS microscopy analysis and the resulting polar plots assign the mitochondrial proteins to their corresponding mitochondrial compartments. The color assigned to each sub-organelle location is based on the annotation frequency (green: 0.75–1; yellow: 0.5–0.75; pink: 0–0.5)

Although for many studies the mapping of protein localization to 14 compartments is sufficient, in theory the MS microscopy allows even better resolution. The resolution of MS microscopy is dependent on the in vivo biotinylation radius (10–50 nm), which then in principle should allow 5–10-fold higher resolution than standard confocal microscope. To benchmark the MS microscopy on sub-organelle analyses, we selected three well-documented mitochondrial proteins and generated a sub-organelle molecular context proteome map of the mitochondrion. The mitochondrion is a double membrane-bound organelle possessing an outer membrane (OMM), an inner membrane (IMM), and mitochondrial matrix within IMM (Fig. 6a). The intermembrane space (IMS) is 10 ∼ 20 nm in diameter, and therefore should allow, for example, almost complete biotinylation and subsequent identification of the proteins in the IMS with MS. The three mitochondrial marker proteins; OMM) outer mitochondrial membrane receptor Tom20 (TOMM20, dark green); IMS) protein SCO1 homolog (SCO1, light green); and matrix) pyruvate dehydrogenase kinase isoform 1 (PDK1, yellow)^43^ were processed through our BioID pipeline to generate the mitochondrial sub-organelle map. This resulted in a mitochondrial sub-organelle reference proteome database consisting of 121 (OMM), 102 (IMS) and 235 (matrix) proteins (Fig. 6b). For testing the generated mitochondrial reference database, we processed additional 13 mitochondrial proteins with our MS microscopy and defined their mitochondrial sub-organelle localization. Using a confocal microscopy we could confirm that all of the proteins are indeed mitochondrial, however it was impossible to obtain more detailed sub-organelle localization information. However, using our MS microscopy we could assign the mitochondrial proteins to the three mitochondrial sub-compartments. Majority of the mitochondrial proteins were localized solely to mitochondria when using the MS microscopy on the whole cell level mainly, and also similarly to a single compartment within the mitochondria when using the MS microscopy on a sub-organelle level (Fig. 6c). Of the tested 13 mitochondrial proteins, eight (MRM1, MGST3, PLRKT, SFXN1, PTH2, COX14, TR61B and AKIP; Supplementary Data 1a) had been analyzed previously using APEX^43^. Our results were in good agreement with the seven of these proteins, however, we also detected COX14 in the IMS, and AKIP in the ‘nucleus’ and ‘chromatin’—location in which AKIP has been reported to also function^44^.

### Defining interaction distances within a protein complex

Others and we have shown that AP-MS offers accurate quantification of complex composition allowing calculations on complex stoichiometry^9,10,45^. With BirA* the labeling radius is limited (circa 10 nm), and it has been used to obtain rough maps of spatial distribution of proteins within structures by reciprocally analyzing BirA*-tagged proteins throughout the structure^15^. As the in vivo biotinylation is enzymatic reaction deriving relational interaction abundances of participants cannot be done. However, it can be reasoned that the more proximal proteins will be more efficiently biotinylated and purified in larger abundances than proteins further away^15,46^. On the AP-MS side this would correlate with the likelihood of more abundant interactors being more direct than low abundant, in which the interaction could be mediated by other proteins and the interaction with the bait would be secondary or tertiary etc. Therefore, by blotting both the BioID and AP-MS data, in theory, we could obtain relative distance of the MAC-tagged bait protein to its interacting protein in a complex. For testing this hypothesis, we selected CDK7 and CDK8 for which we have previously identified successfully quantitative complex compositions^22^.

**Fig. 7:**
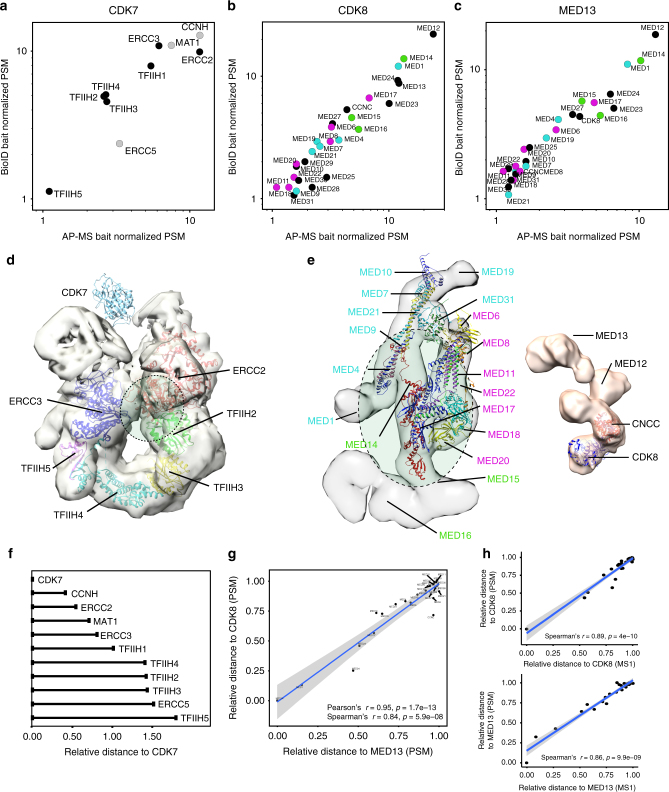
Characterization of interaction distances by integration of MAC-tag data. **a**–**c** Distance based topology of protein complexes. The AP-MS and BioID data was blotted based on the bait normalized prey abundances and the correlated data was used to derive interaction distances for CDK7 and the TFIIH complex, as well as for CDK8 and MED13 with the Mediator complex. The CDK7 formed CAK-complex components are shown in gray and the Mediator complex components assigned to the Head (magenta), the Middle (cyan) and the Tail (green) are color-coded. **d**, **e** The derived interaction distances for CDK7, CDK8 and the MED13 are fitted into EM derived complex structures and suggested fitted interaction surface is shown in green dashed line ellipses. The color-coding in **e** corresponds with **b**, **c**. **f** Relative distances for bait protein and the other complex components can be calculated. (**g**, **h**) The calculated relative distances (using either PSM or MS1 intensity values) derived from the integrated AP-MS and BioID data results to extremely high correlation (Pearson’s and Spearman’s) and p-value as indicated (t-test) for CDK8 and MED13, two neighboring units in the Mediator kinase module

We applied our dual-approach, and with both AP-MS and BioID we could detect the CDK7 interactions with TFIIH core components (Fig. 7a)^47–49^. The size of the TFIIH is estimated to be ~10 nm^47^, which is still within the BirA* biotinylation range and should allow measurement of the CDK7 interaction distances for all of the complex components. Before associating with TFIIH, CDK7 associates and forms cyclin-dependent kinase (CDK)-activating kinase (CAK) complex with two regulatory subunits; cyclin H (CCNH) required for CDK7 activity and with RING finger protein CDK-activating kinase assembly factor MAT1 (MAT) which modulates the substrate specificity of the complex^50^. In agreement, both CCNH and MAT1 are detected as closest to CDK7, followed by ERCC2, ERCC3 and TFIIH1. The ERCC2, TFIIH basal transcription factor complex helicase XPD subunit is the bridge linking the CAK module with TFIIH ring-like core and has been shown to directly interact with TFIIH basal transcription factor complex helicase XPB subunit (ERCC3)^51^ and TFIIH1^52^. CDK7^49^ also has been reported to directly interact with TFIIH1^48^ (Fig. 7d and Supplementary Data 1e)^53,54^. Our results are in line with both hypothesis, the evidence suggesting that TFIIH1 and ERCC3 have short inter-distances as well as that they both are close to the CAK module (Fig. 7d, f). TFIIH2-4 are part of core ring structure of TFIIH complex located adjacent to CDK7, thus having highly similar distance to CDK7 (Fig. 7a, f). Similarly, ERCC5 and TFIIH5 are in longer distances from CDK7, suggesting that they are located on the opposite side of the complex from CAK (Fig. 7a, d, f).

The transcriptional co-activator Mediator complex has more than 30 subunits and is ~30 nm in size^55^, and therefore on the upper detection limits. The Mediator complex is composed of 4 modules, the head^56,57^, the middle^58^, the tail^59^ and the kinase module^60,61^. The evolutionarily conserved and dissociable kinase module is formed by CDK8 together with cyclin C (CCNC), mediator of RNA polymerase II transcription subunit mediator complex subunit (MED) 12 and 13, (Fig. 7e)^56–59^. To test and validate the reproducibility of our approach, in addition to CDK8, we additionally choose MED13 for analysis. Additionally this would allow more accurate prediction of the kinase-module docking surface to the Mediator core complex. To our surprise the overall correlation of CDK8 and MED13 distances from the Mediator core is extremely high (c = 0.95) (Fig. 7b, c, g, h and Supplementary Data 1e), confirming that these two proteins are highly proximal. Based on the analysis the closest Mediator subunits for both CDK8 and MED13 are MED12, MED14, MED1, MED24, MED23, MED17, MED15, MED27, MED16 and MED6/4^59^. This suggests that the kinase module is docking horizontal to the MED14 ranging from RM1 and RM2, the two repeats of a structural domain on MED14.

For additional validation of our approach, we MAC-tagged three components (ARPC1B, ARP2 and ARP3B) of the 220 kDa and 7 seven subunit ARP2/3 complex with high-resolution crystal structure^62^. The obtained relative distances of the three MAC-tagged components to the other components of the ARP2/3 complex are in good agreement with the ARP2/3 structure (Supplementary Figure 11a–c and Supplementary Data 1f).

The CDK7, CDK8, MED13, ARP2/3 examples benchmark another utility of our MAC-tag system and shows that by integration of AP-MS and BioID it is possible to derive information on complex structure, interaction distances and possible distance constraints.

## Discussion

In this study, we developed and optimized an integrated workflow based around MAC-tag, for characterization of the molecular context of many proteins of interest from human cells. This workflow features state-of-the-art affinity purification using Strep-tag to identify and quantify protein-protein interaction and protein complex stoichiometry; identification of transient or close-proximity interactions with BioID; visualization of the bait protein and the proximal interactors with immunofluorescence microscopy; and defining the molecular context with MS microscopy utilizing the reference dataset obtained by identifying proximal interactors for bona fide subcellular localization markers. Additionally our integrated workflow reduces the generation of the required cell lines for AP-MS and BioID to half, and the use of a single affinity reagent simplifies the combinatorial use of both AP-MS and BioID approaches. Additionally, the use of a single affinity reagent facilitates the filtering of the HCIPs (mainly due to the uniform background from the unspecific binders). Other advantages of using Strep-Tactin® in AP-MS include the lower cost and higher binding capacity of Strep-Tactin® compared to antibody beads^63^ (e.g., HA^22^, Myc^10^ and FLAG^20^, as well as the possibility for native elution with biotin instead of on-bead digestion^20^. The possibility for native elution also allows the use of the purified protein complexes for example on enzymatic reactions, such as kinase assays^22^. In addition as the majority of interaction proteomics studies (AP-MS and/or BioID/APEX) are using the biotin-avidin based purification approaches, also the comparison of the obtained results with other publications is easier with using only Strep-Tactin®.

In addition to analyzing the physical and functional interactions formed by 18 cellular localization markers, we used our integrated workflow to map interactions for four kinases (AURKB, CDK7, CDK8 and GSK3B), as well as for two additional localization markers (BET1 and RAB5A). In addition to identifying 539 interactions for these six proteins, we could validate the accuracy of the MS microscopy method for identifying correct cellular localization for these proteins. We also applied the analyses for BioID identified filtered interactions from 7 publications, and derived the cellular localizations for the used baits. The identified localizations were in good agreement with the corresponding reported localizations. This illustrates the general usability of the MS microscopy and the web application, but also suggests that the MS microscopy would be extendable as a communal effort to cover the cell even in greater detail. The cellular signaling state varies between the stimuli, but also with different cell types. Although our MS microscopy resulted in highly similar results using data from three different cell lines, the biological variation and cellular heterogeneity needs to be taken into consideration with MS microscopy as with any biological experiment.

Furthermore, we could show with an exogenous protein, AOX that our MS microscopy identifies AOX to localize to mitochondria. Additional analysis shows that AOX localizes to inner mitochondrial membrane from the mitochondrial matrix side and is in close-proximity with Complex II. Our findings validate, the functionally suggested vicinity of AOX with Complex II. We further extended our analysis on sub-organelle level with mitochondria, by addition of three additional markers, TOMM22 for OMM, SCO1 for IMS and PDK1 for the mitochondrial matrix. Using this sub-organelle reference proteome map, we applied MS microscopy on 13 mitochondrial proteins and could identify their sub-organelle locations, whereas the confocal microscopy (93×) failed to do so.

Identifying the complex components in a stoichiometry fashion has been shown to be possible with affinity purification mass spectrometry^9,10^. However, obtaining any further spatial information of the complex formation has only been possibly in combination with XL-MS^18^. We could now show with the TFIIH and Mediator complex as model complexes, that by utilizing both the AP-MS and BioID approaches we can obtain relative interaction distances for proteins in a complex. Based on the interaction distances it is possible to obtain an estimate for the interaction surfaces for proteins or structures, such as with the kinase submodule of the Mediator complex. We agree that deriving the interaction distances might only be applicable when using single affinity reagent (Strep-Tactin®) for both the AP-MS and BioID.

In summary, our study showed that the integrated workflow and the reference molecular context proteome map generated here, allows an easy way to probe the molecular localization of protein of interest, and additionally an online resource of our BioID based MS microscopy approach is available at www.biocenter.helsinki.fi/bi/protein/msmic. We also showed that it is also usable for existing BioID datasets and can be expanded (with additional localizations or cell types) once more data comes available. The molecular image obtained from the MS microscopy analysis considers the weights of interactors and provides more dynamic localization information at the molecular level. The developed MAC-tag and the integrated approach should empower, not only the interaction proteomics community, but also cell/molecular/structural biologists, with an experimentally proven integrated workflow for mapping in detail the physical and functional interactions and the molecular context of proteins in human cells.

## Methods

### Generation of MAC-tag Gateway® destination vectors

To generate Gateway compatible destination vectors, plasmids containing the tags (C-terminal: StrepIII/HA/BirA*, N-terminal: BirA*/HA/StrepIII) were synthesized by GeneArt®, Life Technologies. These were digested with restriction enzymes and inserted into N-terminal: pcDNA5/FRT/TO/StrepIII/HA/GW^64^ or C-terminal: pcDNA5/FRT/TO/StrepIII/HA/GW^2^ in which entire StrepIII/HA tag was removed. All the Gateway compatible entry clones, which contain subcellular marker gene of interested, were from Human ORFeome collection. The MAC-tag constructs are made available via Addgene.org.

### Immunofluorescence

HeLa cells (American Type Culture Collection, ATCC, CCL-2) were transfected with vectors containing MAC-tagged gene of interest and cultured either with or without supplemental biotin. Bait proteins were detected with anti-HA antibody (Biolegend, MMS-101R, dilution 1:500), followed by Alexa Fluor 488-conjugated secondary antibody (Thermo Fisher Scientific, A-11001, 1:800). Biotinylated proteins were detected with Alexa Fluor 594 streptavidin (Thermo Fisher Scientific; S11227, 1:800). DAPI staining was used to determine the nuclei. Selected endogenous proteins were detected with specific antibodies (Supplementary Data 1a) and subsequently with Alexa Fluor 594 -conjugated anti-rabbit antibody (Thermo Fisher Scientific, A11012, dilution 1:800). Wide-field fluoresce microscope (Leica, Leica DM6000, Welzlar, Germany) with HCXPL APO 63×/1.40–0.60 oil objective was used to image the samples. For imaging sub-mitochondrial proteins, confocal microscopy (Leica TCS SP8 STED, Leica) with HC PL APO 93×/1.30 motCORR glycerol object was used. The image files were processed with LAS X (Leica), and ImageJ softwares.

### Cell culture

For generation of the stable cell lines inducibly expressing the MAC-tagged versions of the baits, Flp-In™ T-REx™ 293 cell lines (Invitrogen, Life Technologies, R78007, cultured in manufacture’s recommended conditions) were co-transfected with the expression vector and the pOG44 vector (Invitrogen) using the Fugene6 transfection reagent (Roche Applied Science). Two days after transfection, cells were selected in 50 μg ml^*−*1^ streptomycin and hygromycin (100 μg ml^−1^) for 2 weeks, and then the positive clones were pooled and amplified. Stable cells expressing MAC-tag fused to green fluorescent protein (GFP) were used as negative controls and processed in parallel to the bait proteins.

Each stable cell line was expanded to 80% confluence in 20 × 150 mm cell culture plates. Ten plates were used for AP-MS approach, in which 1 μg ml^−1^ tetracycline was added for 24 h induction, and 10 plates for BioID approach, in which in addition to tetracycline, 50 μM biotin was added for 24 h before harvesting. Cells from 5 × 150 mm fully confluent dishes (~5 × 10^7^ cells) were pelleted as one biological sample. Thus, each bait protein has two biological replicates in two different approaches. Samples were snap frozen and stored at −80 °C.

Human osteosarcoma cell line U-2 OS (ATCC, HTB-9) and prostate cancer cell line DU145 (ATCC, HTB-81) were routinely maintained in ATCC-recommended conditions. For transient transfections, DU-145 or U-2 OS cells were seeded 24 h before transfection to 7 × 150 mm cell culture plates. Transient transfections for U-2 OS were conducted using DreamFect Gold transfection reagent (OZ Biosciences, Marseille, France), and for DU-145 with Helix-IN™ (FP29, OZ Biosciences) according to the manufacturer’s instructions. The in vivo biotinylation was activated by addition of biotin 24 h post-transfection to a final concentration of 50 μM. Cells from the 7 × 150 mm cell culture plates (~7 × 10^7^ cells) were collected and snap frozen. Analyses of the baits were performed as two biological replicates.

### Affinity purification of the interacting proteins

For AP-MS approach, the sample was lysed in 3 ml of lysis buffer 1 (0.5% IGEPAL, 50 mM Hepes, pH 8.0, 150 mM NaCl, 50 mM NaF, 1.5 mM NaVO_3_, 5 mM EDTA, supplemented with 0.5 mM PMSF and protease inhibitors; Sigma).

For BioID approach, Cell pellet was thawed in 3 ml ice-cold lysis buffer 2 (0.5% IGEPAL, 50 mM Hepes, pH 8.0, 150 mM NaCl, 50 mM NaF, 1.5 mM NaVO_3_, 5 mM EDTA, 0.1% SDS, supplemented with 0.5 mM PMSF and protease inhibitors; Sigma). Lysates were sonicated, treated with benzonase.

Cleared lysate was obtained by centrifugation and loaded consecutively on spin columns (Bio-Rad) containing lysis buffer 1 prewashed 200 μl Strep-Tactin beads (IBA, GmbH). The beads were then washed 3 × 1 ml with lysis buffer 1 and 4 × 1 ml with wash buffer (50 mM Tris-HCl, pH 8.0, 150 mM NaCl, 50 mM NaF, 5 mM EDTA). Following the final wash, beads were then resuspended in 2 × 300 μl elution buffer (50 mM Tris-HCl, pH 8.0, 150 mM NaCl, 50 mM NaF, 5 mM EDTA, 0.5 mM Biotin) for 5 mins and eluates collected into an Eppendorf tubes, followed by a reduction of the cysteine bonds with 5 mM Tris(2-carboxyethyl)phosphine (TCEP) for 30 mins at 37 °C and alkylation with 10 mM iodoacetamide. The proteins were then digested to peptides with sequencing grade modified trypsin (Promega, V5113) at 37 °C overnight. After quenching with 10% TFA, the samples were desalted by C18 reversed-phase spin columns according to the manufacturer’s instructions (Harvard Apparatus). The eluted peptide sample was dried in vacuum centrifuge and reconstituted to a final volume of 30 μl in 0.1% TFA and 1% CH_3_CN.

### Liquid chromatography–mass spectrometry (LC-MS)

Analysis was performed on a Q-Exactive mass spectrometer using Xcalibur version 3.0.63 coupled with an EASY-nLC 1000 system via an electrospray ionization sprayer (Thermo Fisher Scientific). In detail, peptides were eluted and separated with a C18 precolumn (Acclaim PepMap 100, 75 μm x 2 cm, 3 μm, 100 Å, Thermo Scientific) and analytical column (Acclaim PepMap RSLC, 75 μm × 15 cm, 2 μm, 100 Å; Thermo Scientific), using a 60 min buffer gradient ranging from 5 to 35% buffer B, followed by a 5 min gradient from 35 to 80% buffer B and 10 min gradient from 80 to 100% buffer B at a flow rate of 300 nl min^−1^ (buffer A: 0.1% formic acid in 98% HPLC grade water and 2% acetonitrile; buffer B: 0.1% formic acid in 98% acetonitrile and 2% water). For direct LC-MS analysis, 4 μl peptide samples were automatically loaded from an enclosed cooled autosampler. Data-dependent FTMS acquisition was in positive ion mode for 80 min. A full scan (200–2000 m z^*−*1^) was performed with a resolution of 70,000 followed by top10 CID-MS^2^ ion trap scans with resolution of 17,500. Dynamic exclusion was set for 30 s. Acquired MS^2^ spectral data files (Thermo.RAW) were searched with Proteome Discoverer 1.4 (Thermo Scientific) using SEQUEST search engine of the selected human component of UniProtKB/SwissProt database (http://www.uniprot.org/, version 2015–09). The following parameters were applied: Trypsin was selected as the enzyme and a maximum of 2 missed cleavages were permitted, precursor mass tolerance at ±15 ppm and fragment mass tolerance at 0.05 Da. Carbamidomethylation of cysteine, was defined as static modifications. Oxidation of methionine and biotinylation of lysine and N-termini were set as variable modifications. All reported data were based on high-confidence peptides assigned in Proteome Discoverer with FDR<1%).

### Identification of the HCIs

Significance Analysis of INTeractome (SAINT)-express version 3.6.0^65,66^ and Contaminant Repository for Affinity Purification (CRAPome, http://www.crapome.org/)^67^ were used as statistical tools for identification of specific high-confidence interactions from our AP-MS data. 16 GFP control runs (8 N-terminal MAC-GFP and 8 C-terminal MAC-GFP) were used as control counts for each hit and the final results only considering proteins with SAINT score ≥ 0.73. This corresponds to an estimated protein-level Bayesian FDR of <0.05. Furthermore, we used the CRAPome database with a cutoff frequency of ≥20% (≥82) except the average spectral count fold change ≥3 was set for assigning HCIs.

### Clustering analysis

Prey protein frequency count matrix was generated using DAVID gene functional classification tool to provide the gene ontology (GO) terms (domains, biological process and molecular function). The p-values associated with each annotation terms has *p* < 0.01(by a modified Fisher’s exact test). Hierarchical cluster was performed by centered correlation (both baits and interactors; average linkage) using Cluster 3.0 and the clusters were visualized with Tree View 1.1.6 and the matrix2png web server (http://www.chibi.ubc.ca/matrix2png/).

### Networks and maps

Protein interaction networks are constructed from SAINT data that were imported into Cytoscape 3.2.1^68^. The known prey-prey interaction data were obtained from PINA2 database (http://omics.bjcancer.org/pina/)

### MS microscopy database construction

The high-confidence interacting proteins (HCIPs) obtained from previous filtering steps were sorted according to the corresponding bait protein localization information to build the reference database, containing the following localization information: peroxisome, microtubule, endosome (combined: early, late and recycling endosome), proteasome, nuclear envelope, Golgi (combined: *trans*-Golgi and *cis*-Golgi), lysosome, nucleolus, plasma membrane, endoplasmic reticulum, mitochondria, centrosome, chromatin, exosome. To elucidate sub-organelle localization (OMM, IMS and matrix) within mitochondria, three mitochondrial bait proteins were used.

### Score calculation

Final localization scores for all localization groups of a given bait of interest were calculated by first dividing the sum of the normalized peptide-spectrum match (PSM) values of the interactors that matched to the bait of interest and a localization group by the sum of PSM values of all bait interactors that matched any localization group. This was then multiplied by the sum of PSM values of interactors of the localization group that matched the bait interactors of interest divided by the sum of PSM values of all interactors of the localization group. The PSMs were bait normalized, i.e., the PSM of each interactor was divided by the PSM of the bait. The score reflects subcellular localization by numerically describing the similarity of the subcellular environment and the time spent there between the bait of interest and each localization group.

### Results visualization

The MS microscopy analyses are presented as polar plots created with an in-house python script, where the circle has been equally divided into 14 sectors, each sector representing one specific subcellular location. For the sub-organelle localization analysis, three sectors representing matrix, IMS and OMM are shown in the plot. Differently colored sector areas indicate the possible location score of the query bait, with scores between 0 and 0.5 marked in red, between 0.5 and 0.75 in yellow, and 0.75 and 1 in green.

### Online interface

We have developed a web application (R-shiny; http://www.biocenter.helsinki.fi/bi/protein/msmic) for visualization of protein localizations by MS microscopy. A user can upload an input file (after SAINT and CRAPome filtering) and visualize the bait protein’s dynamic localization. The polar plot as well as a parsed data matrix can also be downloaded.

### Determining relative intramolecular distances of complexes

The PSM values from both the BioID and AP-MS approaches were first averaged from the replicate samples and the averaged PSMs from the preys were normalized by dividing them by the averaged PSMs of the baits. The normalized PSMs were then used to calculate the relative Euclidean distance between the baits and the preys using the formula $\sqrt {({\mathrm{PSM}}_{{\mathrm{Prey}}}^{{\mathrm{BioID}}} - {\mathrm{PSM}}_{{\mathrm{Bait}}}^{{\mathrm{BioID}}})^2 + ({\mathrm{PSM}}_{{\mathrm{Prey}}}^{{\mathrm{AP}} - {\mathrm{MS}}} - {\mathrm{PSM}}_{{\mathrm{Bait}}}^{{\mathrm{AP}} - {\mathrm{MS}}})^2}$ in an in-house R script. These values (in log10 scale) were also used to create the scatter plots. Pearson’s and Spearman’s correlations were used to assess the correlations between the relative distance measures of the CDK8 and MED13 baits (the correlation coefficients and their associated p-values are shown). MaxQuant (version 1.6)^69^ was used to obtain the parent-ion mass (MS1) information.

### Crystal structure and CryoEM docking

The human core TFIIH crystal structure (Protein Data Bank (PDB): 5IVW) was docked into cryoEM reconstructions described by He et al. (Electron Microscopy Data Bank (EMDB): 8131)^48^. The crystal structure of human CDK7 (Protein Data Bank: 1UA2)^49^ was simply placed in the possible interaction direction using Chimera. The human CDK8 crystal structure (PDB: 3RGF)^61^ was docked into cryoEM reconstructions described by Tsai et al. (EMDB: 5588)^60^. The mediator complex structure for head module (PDB:4GWP^57^, middle module^58^, tail module(5U0S)^59^ were docked into cyroEM reconstructions described by Tsai et al. (EMDB:2634)^56^. Crystal structure of Arp2/3 complex (PDB: 4JD2)^62^ was showing in surface representation.

### Data availability

The MS data for all the 518 runs are available at the PeptideAtlas raw data repository under the accession code PASS01076 (see also Supplementary Data 1b). The protein interactions from this publication have been submitted to the IMEx consortium (http://www.imexconsortium.org) through IntAct^70^ and assigned the identifier IM-26301. Results of using MS microscopy on published BioID datasets are available at http://www.biocenter.helsinki.fi/bi/protein/msmic/example.pdf. Other data that support this study are available from the corresponding author upon reasonable request.

## Electronic supplementary material

Supplementary Information(PDF 6247 kb)

Peer Review File(PDF 85 kb)

Description of Additional Supplementary Files(PDF 165 kb)

Supplementary Data 1(XLSX 794 kb)

Supplementary Data 2(XLSX 241 kb)

Supplementary Data 3(XLSX 15 kb)
