# Supplementary material for: An AP-MS- and BioID-compatible MAC-tag enables comprehensive mapping of protein interactions and subcellular localizations
Source: Nat Commun. 2018 Mar 22;9:1188. doi: 10.1038/s41467-018-03523-2 (PMC5864832; doi:10.1038/s41467-018-03523-2)
Supplement: Supplementary file 1 — Supplementary Information(PDF 6247 kb) [file 41467_2018_3523_MOESM1_ESM.pdf]

An AP-MS- and BioID-compatible MAC-tag enables comprehensive mapping of protein interactions and subcellular localizations

Liu *et al*.

Supplementary Figures

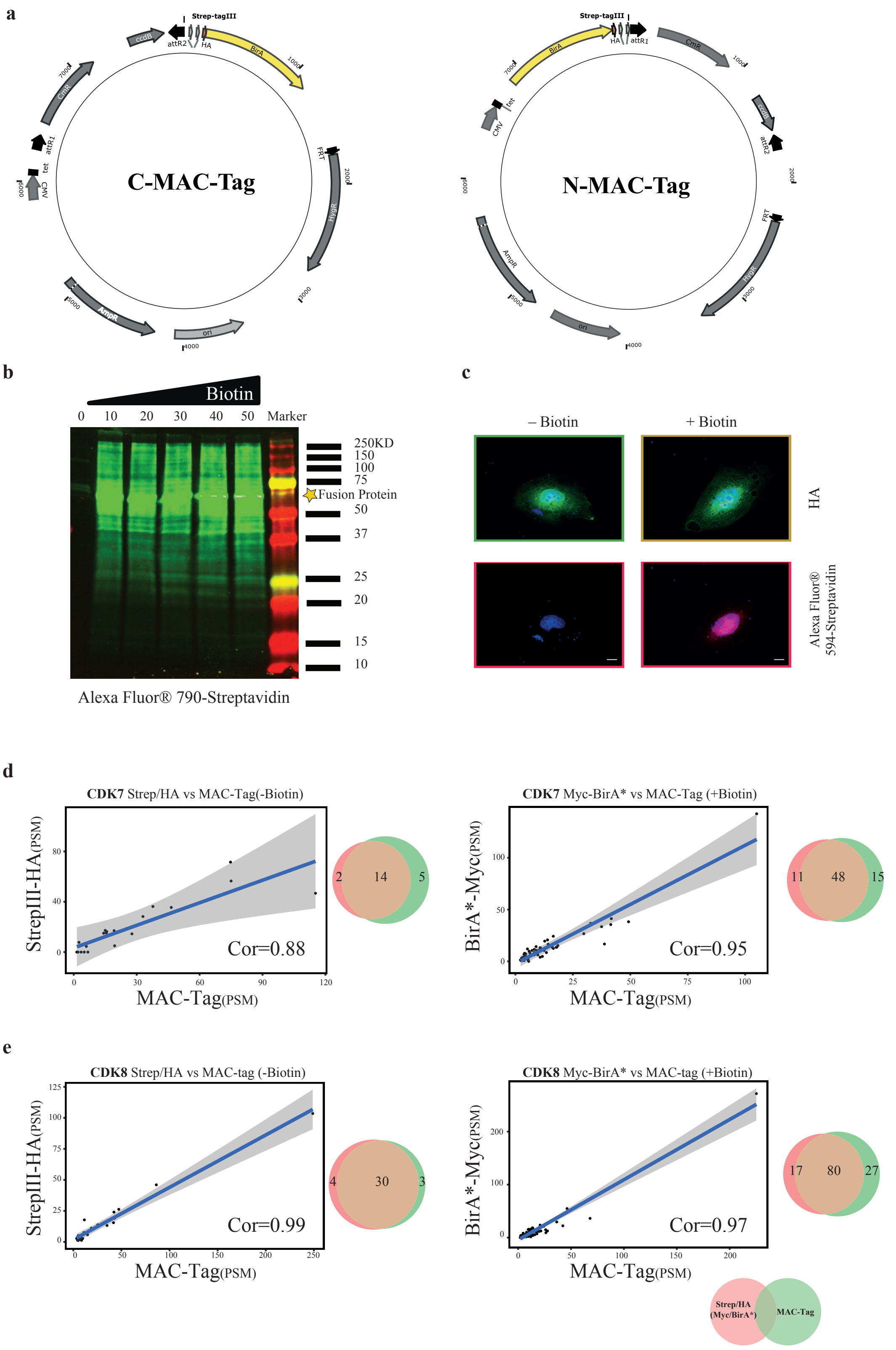

Supplementary Figure 1. Features of the MAC-tag. (a) Plasmid maps are graphical representation of the MAC-tag vectors. (b) The western blot shows biotinylated proteins of MAC-tagged GFP cell lysate with biotin concentration gradient (0-50 µM) in culture medium by Alexa Fluor®594-conjugated streptavidin. (c) Im-munofluorescence analysis shows no detectable biotinylation in untreated sample and significant activation of the biotinylation with 10 µM biotin and peaking at 50 µM concentration (anti-HA: green; Alexa Fluor®594-conjugated streptavidin: red; DAPI: blue. Scale bar: 10 µm). (d-e) There is strong correlation (Pearson's) between MAC-tag AP-MS (no biotin) & StrepIII-HA and MAC-tag BioID (with biotin) & BirA*-Myc. A scatter plot shows 95% confi dence interval of correlation between MAC-tag data and the data from StrepIII-HA & BirA*-Myc alone (p-value < 0.01, t-test). Venn's diagrams illustrate the overlap comparison of the identfi ed HCIPs be-tween the different tags.

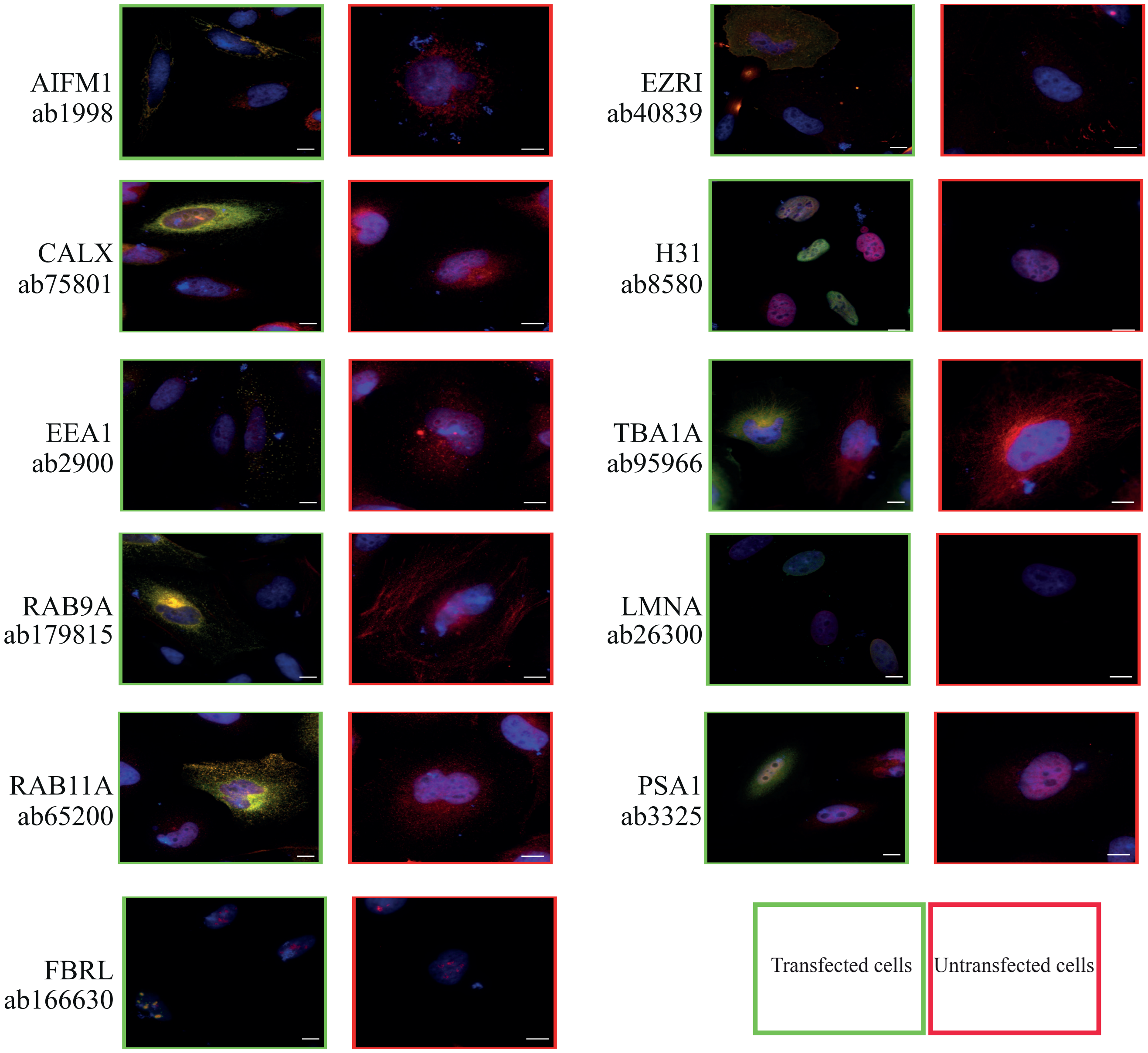

Supplementary Figure 2. Immunofluorescence analysis shows highly similar localization of the MAC-tagged proteins with their corresponding endogenous proteins.The localization of eleven MAC-tagged proteins were compared to their endogenous counterparts. The MAC-tagged proteins were visualized with anti-HA im-munostaining (green) and the corresponding endogenous proteins with protein specific antibodies and Alexa Fluor®594-conjugated secondary antibody (red). The nucleus was stained with DAPI (blue). Scale bar: 10 µm.

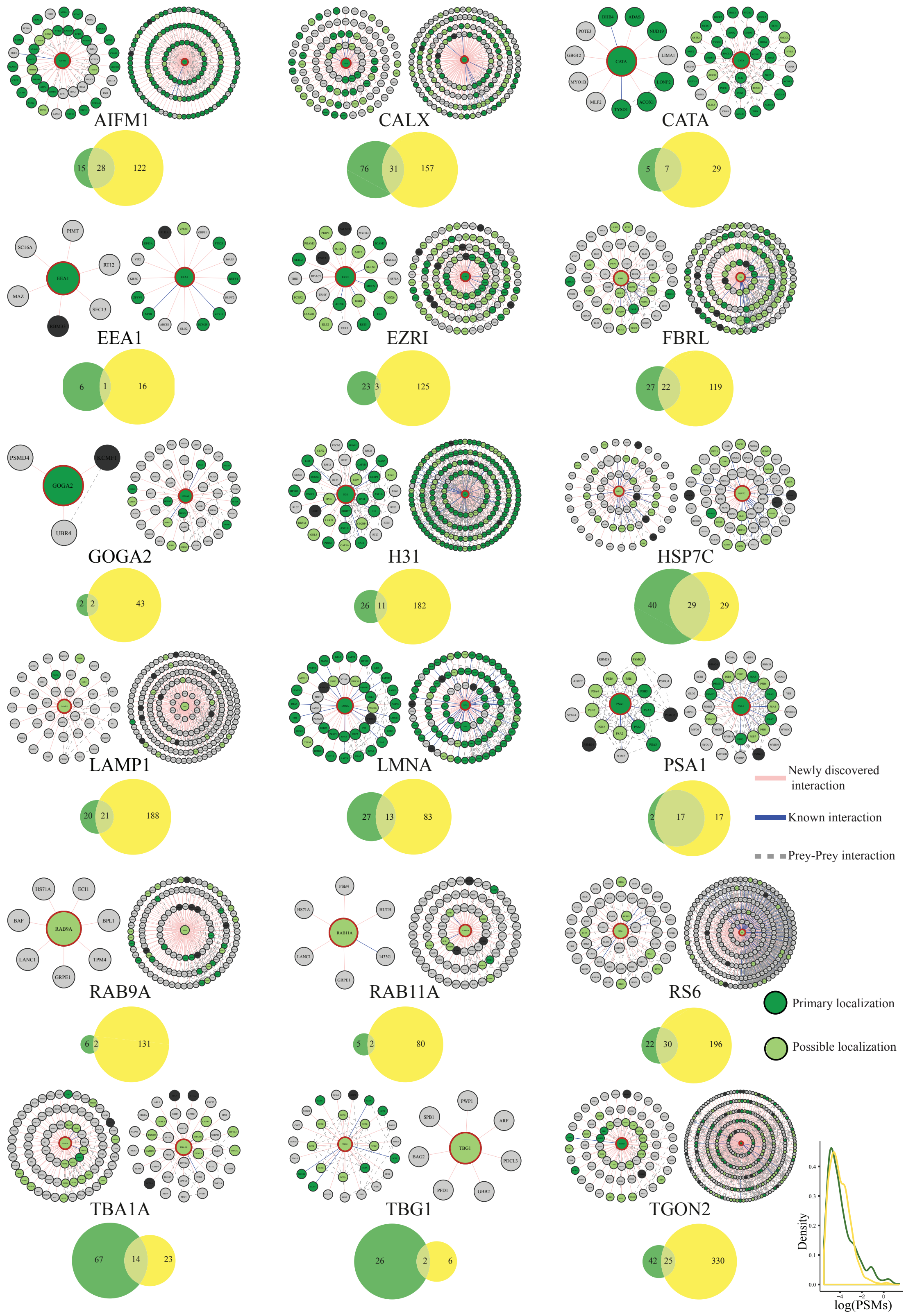

Supplementary Figure 3. Overview of high-confidence interactions of 18 *bona fide* cellular localization markers. The individual distribution of interactions detected with of AP-MS and BioID approaches with MAC-tagged 18 *bona fide* cellular localization markers. The newly identified interactions are presenting in pink lines and the blue line represents the known interaction. Prey-prey interactions represent via dash lines. The nodes are color-coded based on the localization rank obtained from the CellWhere database (key: dark green = pri-mary cellular localization for the corresponding protein, light green = possible localization, grey = different or localization assigned for the protein). Venn's diagram compares the number of interactions between AP-MS and BioID -methods, overlap showing the number of common interactions. Lower right inset: The distribution of the bait normalized AP-MS (green) and BioID (yellow) values for the HCIs (ratio-to-the-bait) are highly comparable.

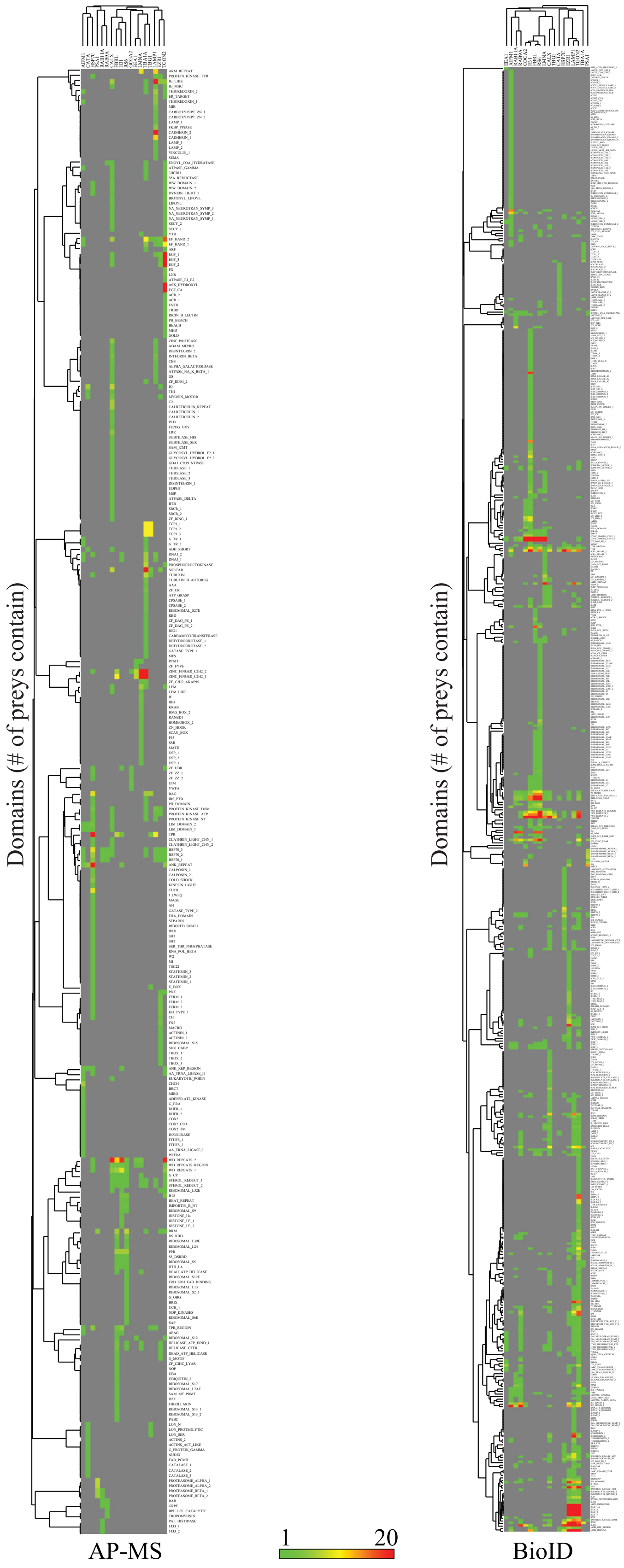

Supplementary Figure 4. Hierarchical clustering the domain containing of the HCIPs of 18 localization markers. The DAVID bioinformatics recourses 6.8 database (https://david.ncifcrf.gov/) were used for interaction domain containing analysis to generate the value count matrix for clustering (P<0.01, by a modified Fisher's exact test). The color intensities indicate the sum of domain containing count.

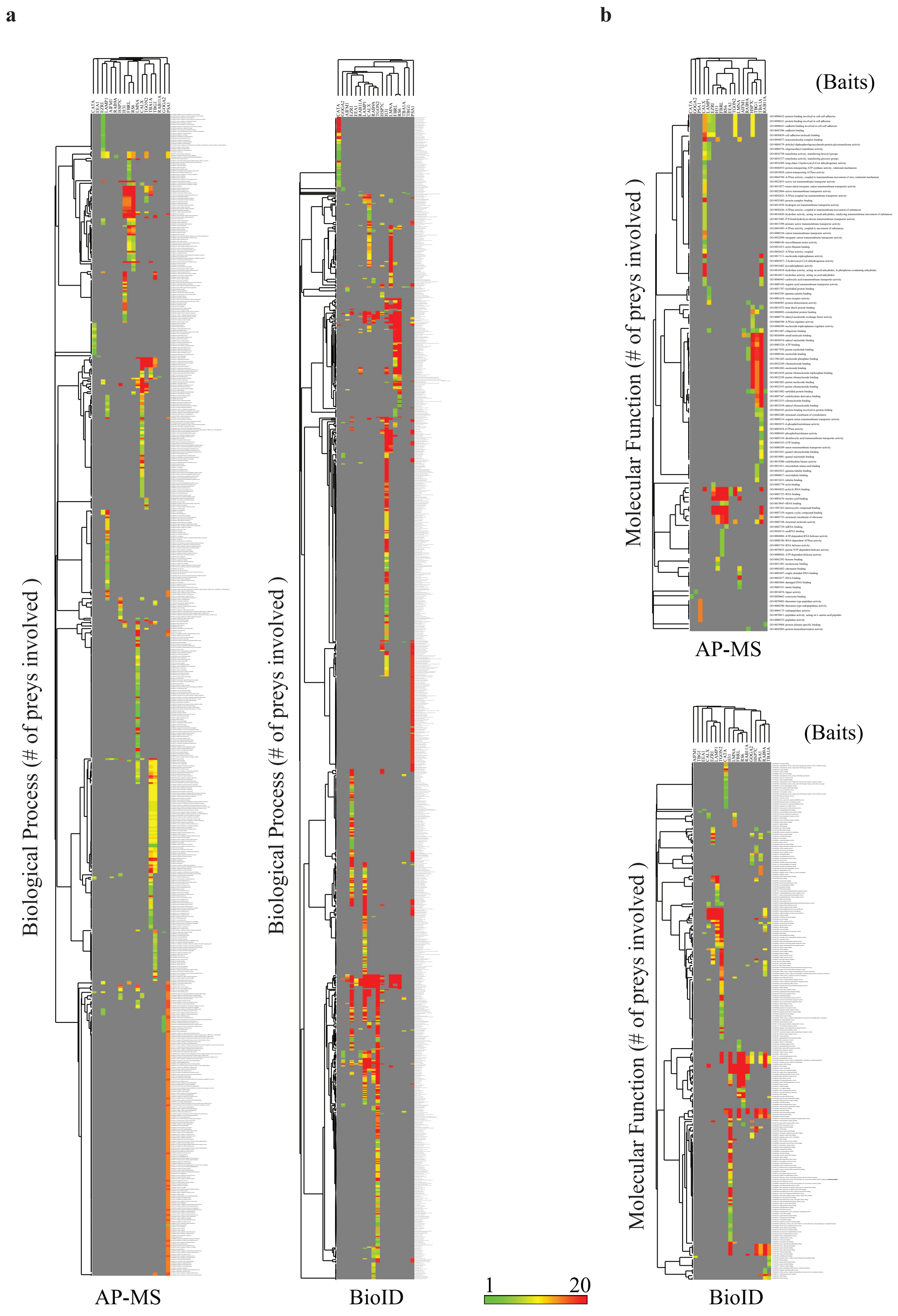

Supplementary Figure 5. Gene Ontology analysis of interaction contexts of 18 localization markers. The DAVID bioinformatics recourses 6.8 database (https://david.ncifcrf.gov/) are used for GO term analysis to generate the value count matrix for clustering with a p-value cut-off of P < 0.01(by a modifi ed Fisher's exact test). The hierarchically clustered heatmap including: biology process (a) and molecular function (b). The color intensities indicate the sum of preys of corresponding GO term. Hierarchal clustering was used to generate the cluster dendrogram.

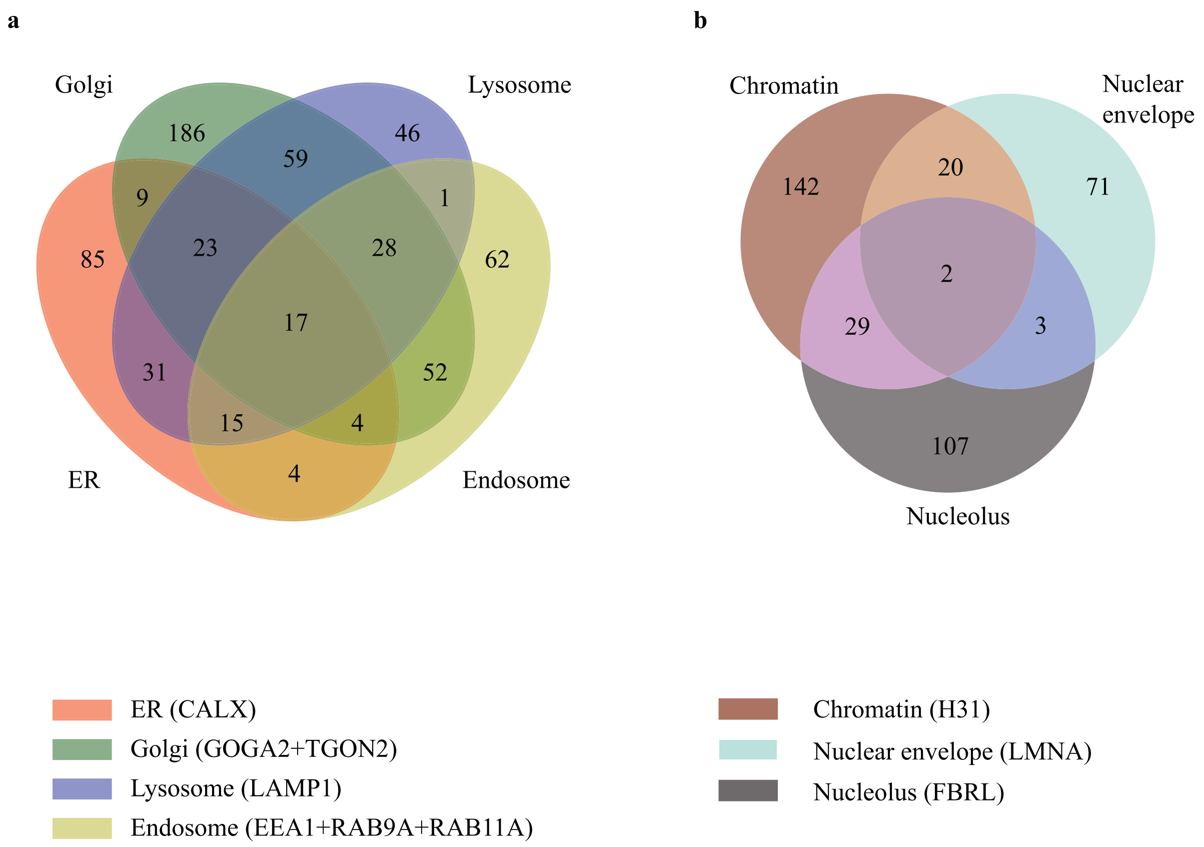

Supplementary Figure 6. Inter-relation of different subcellular organelles. Venn's diagram compares the number of identified HCIPs from different subcellular compartments to the biological relationship between different organelles. (a) BioID identified proteins that are trafficking among Golgi, lysosome, endosome and ER are shown. (b) BioID identified proteins trafficking among nucleolus, NE and chromatin are shown.

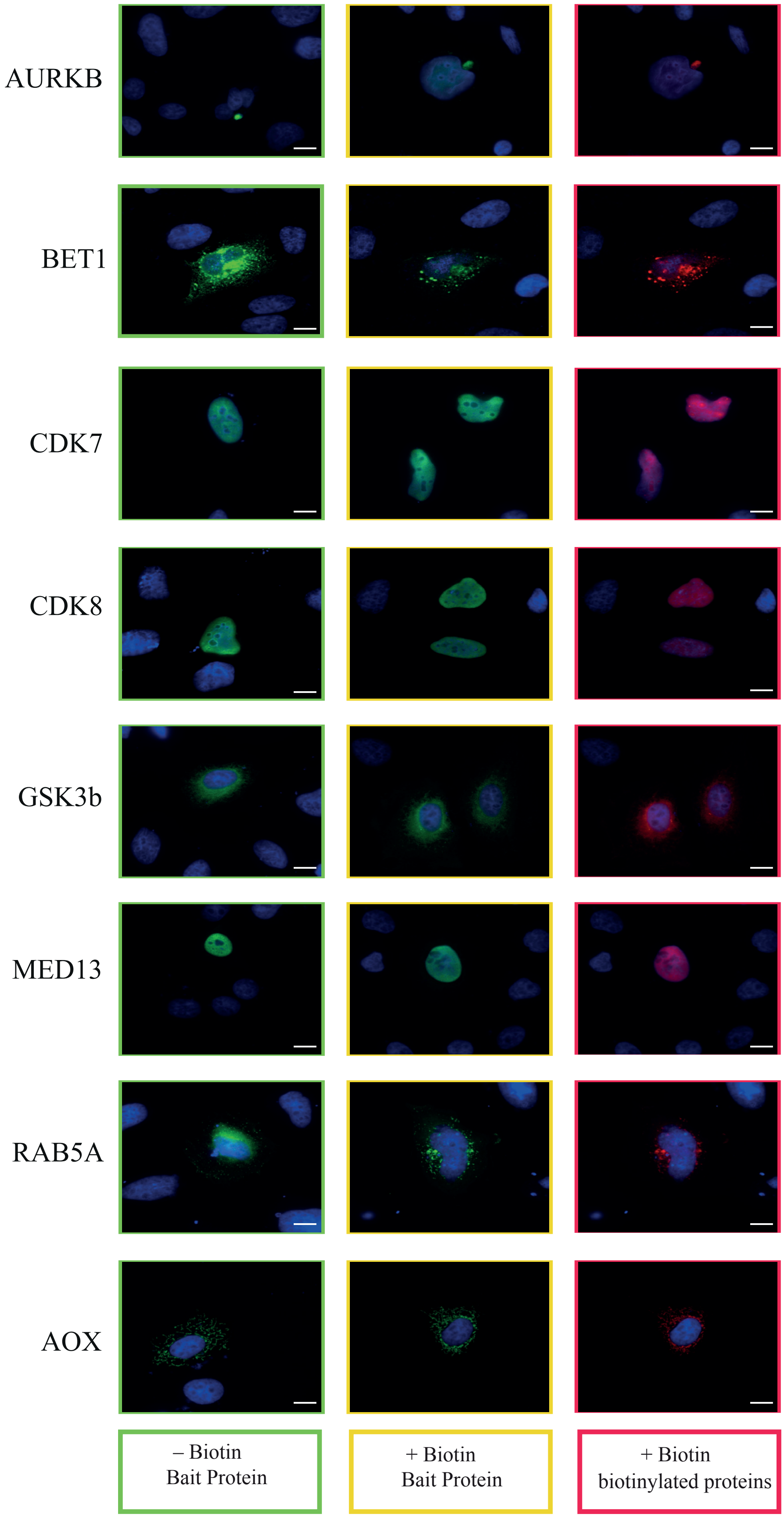

Supplementary Figure 7. Validation of protein localization and proximal biotinylation with immunofluorescence. Fluorescence microscopy (HCX PL APO 63x/1.40 – 0.60 oil (0.10 mm) was applied to observe the MAC-tagged proteins that have been monitored by MS-microscopy (Fig. 4). These tagged baits are visualized with anti-HA immunostaining (green), DAPI (blue) and the in vivo biotinylated interactors are staining with Alexa Fluor®594-conjugated streptavidin (red), Scale bar: 10 µm.

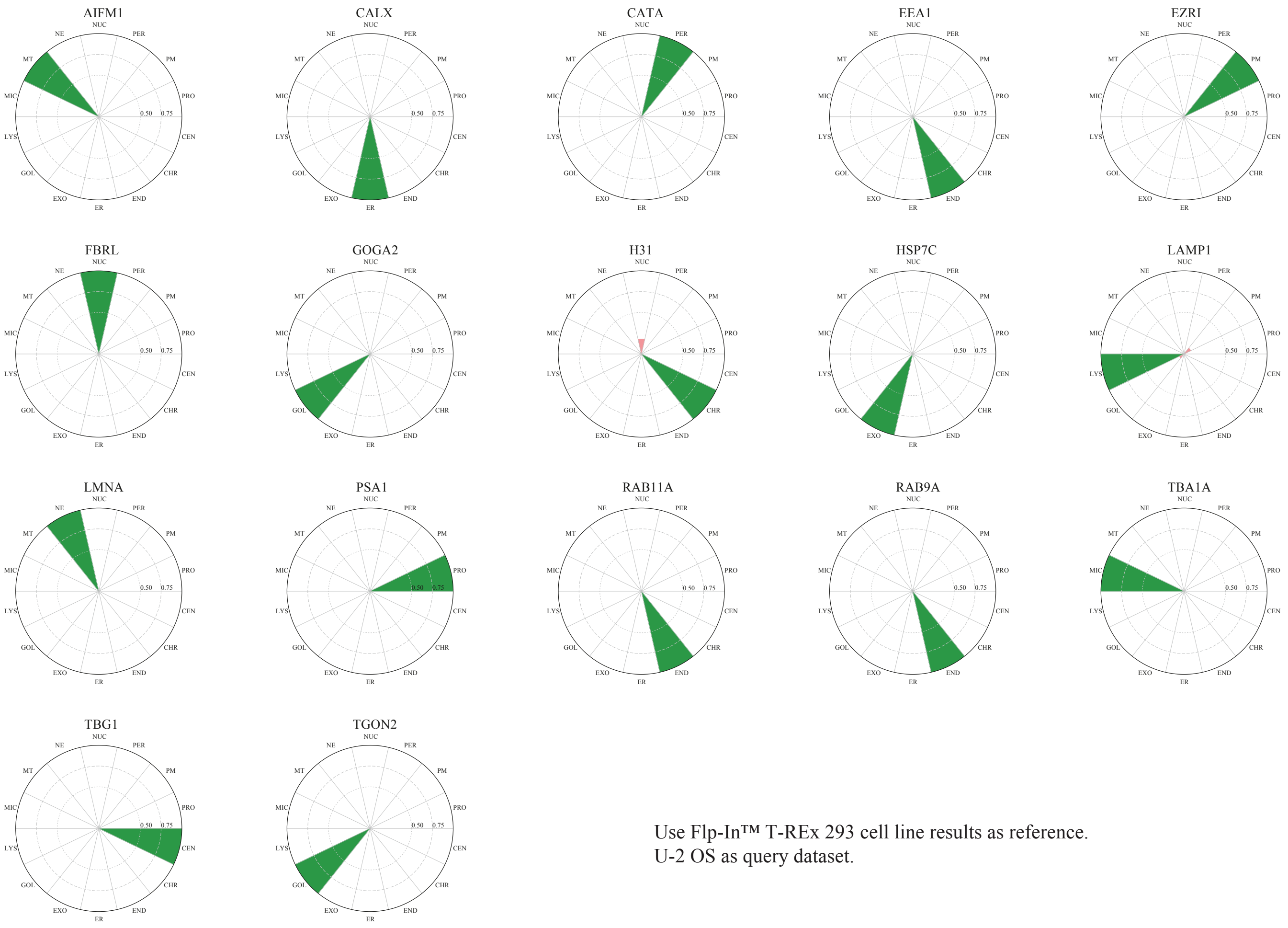

Supplementary Figure 8. MS-microscopy analysis of the 17 cellular localization markers transiently transfected to the U-2 OS cells.The 17 cellular localization markers were transiently transfected to the U-2 OS cells and the MS-microscopy analysis performed against the Flp-In™ T-REx 293 as a reference. The color assigned to each of the localization is based on the annotation frequency (pink: 0-0.5; yellow: 0.5-0.75; green: 0.75-1).

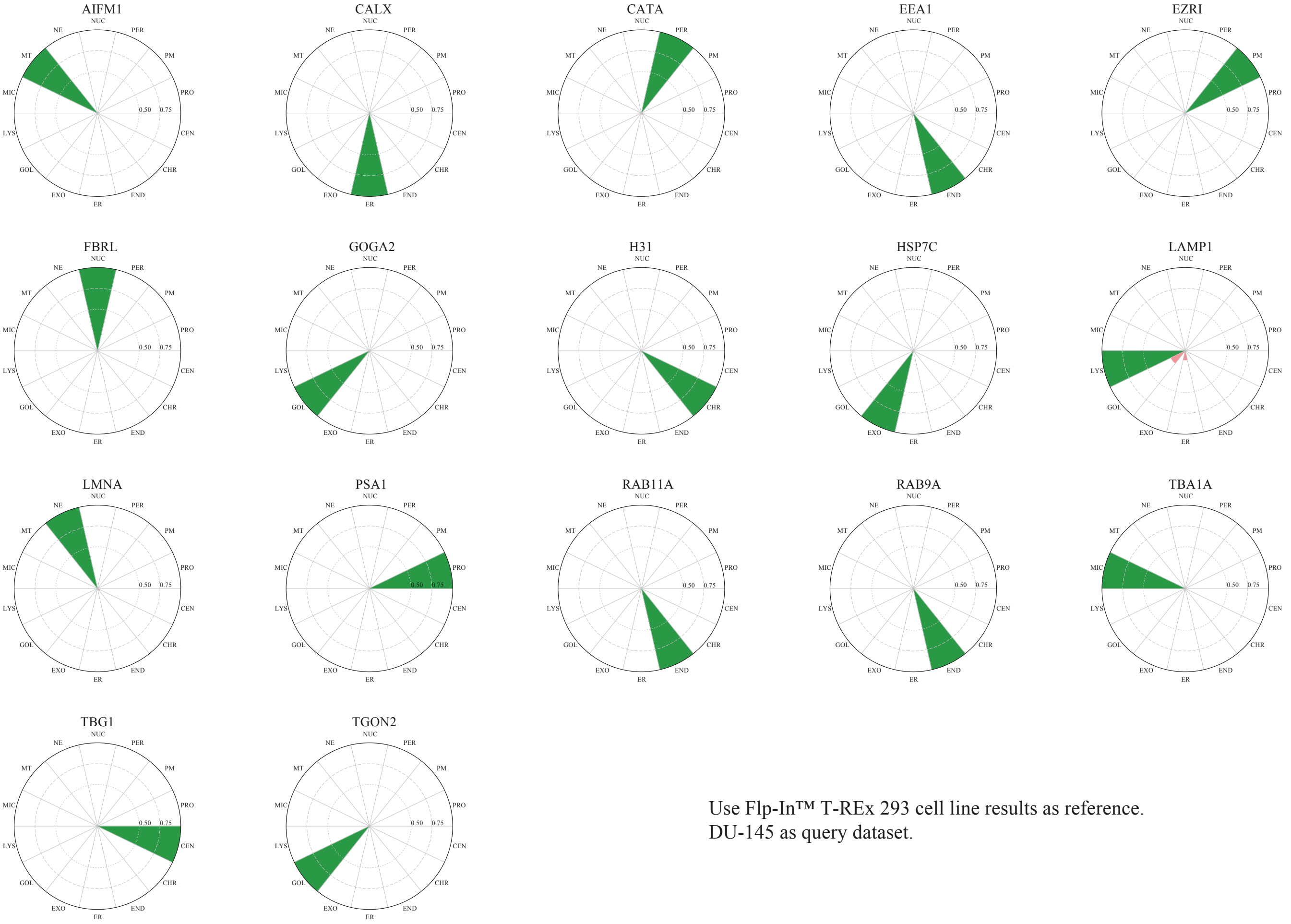

Supplementary Figure 9. MS-microscopy analysis of the 17 cellular localization markers transiently transfected to the DU-145 cells.The 17 cellular localization markers were transiently transfected to the DU-145 cells and the MS-microscopy analysis performed against the Flp-In™ T-REx 293 as a reference. The color assigned to each of the localization is based on the annotation frequency (Pink: 0-0.5; Yellow: 0.5-0.75; Green: 0.75-1).

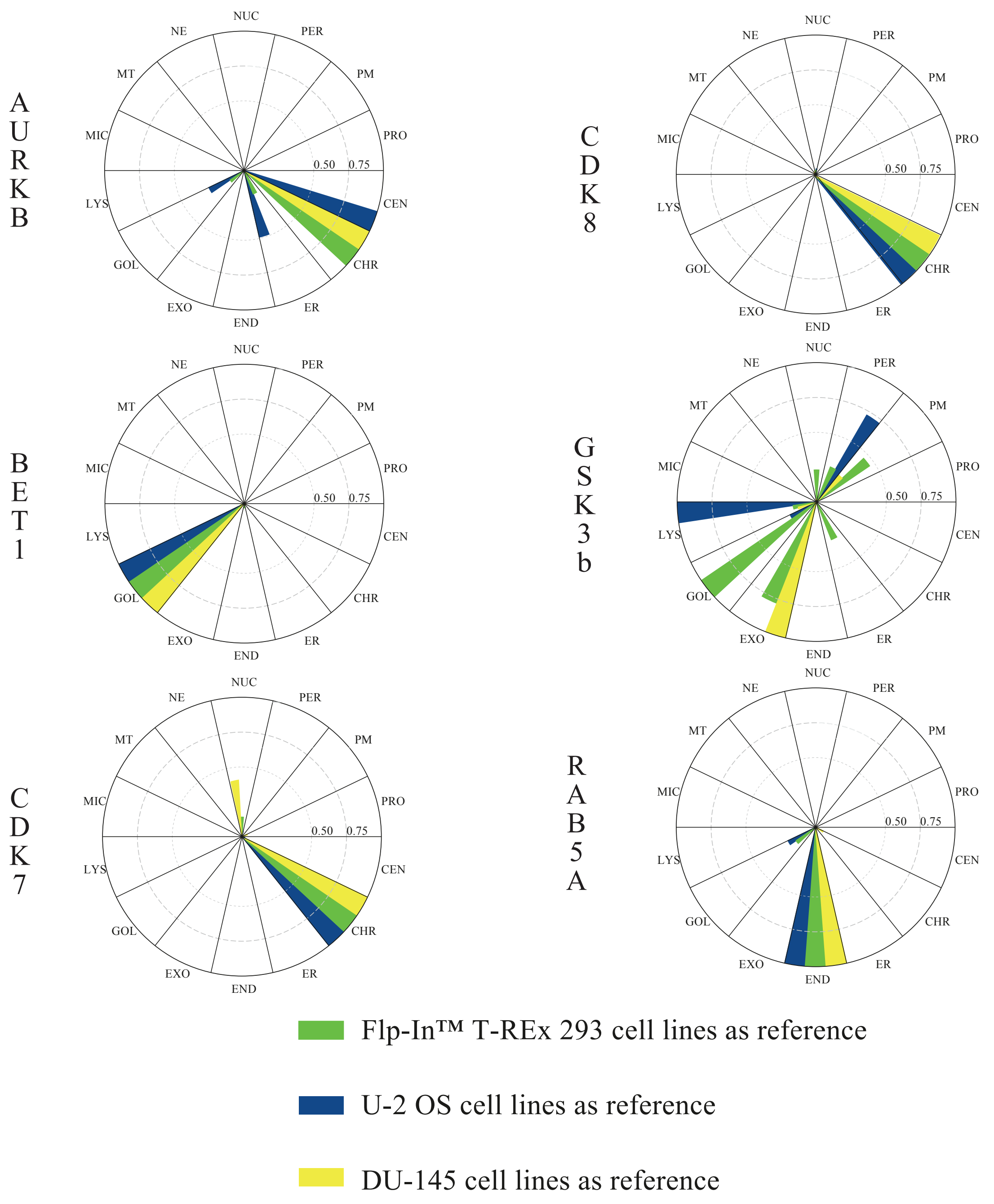

Supplementary Figure 10. MS-microscopy analysis using reference molecular context maps from three different cell lines. The MS-microscopy analyses for six proteins were performed using the reference molecular context maps from Flp-In™ T-REx 293 (green), U-2 OS (blue) and DU-145 (yellow) cell lines. The polar plot shows the location of each queried protein.

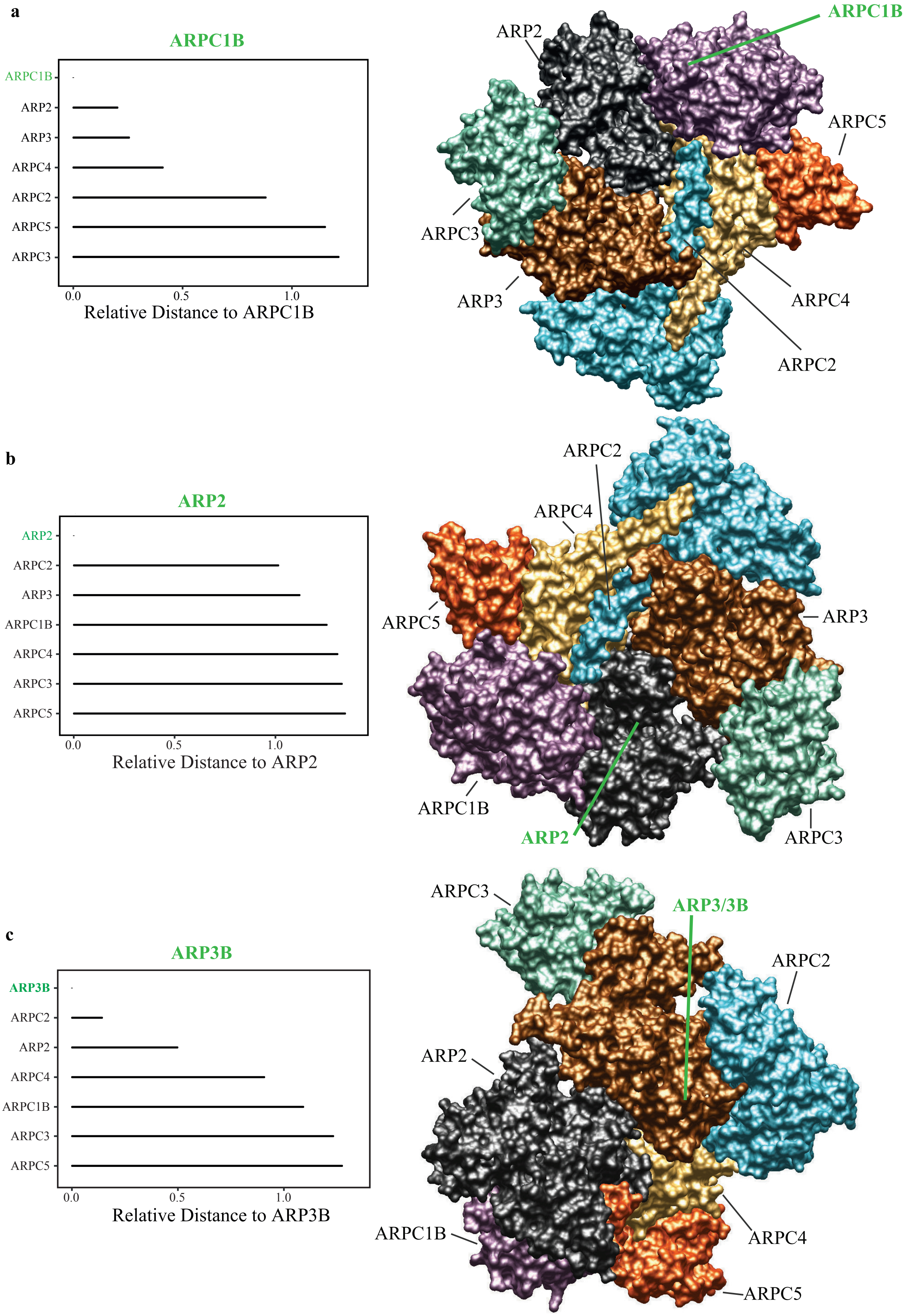

Supplementary Figure 11. Interaction distances estimated using integration of MAC-tag data for three components of the Arp2/3 complex match well with the Arp2/3 complex structure. (a-c) The relative distances for ARPC1B, ARP2 and ARP3B, to the other Arp2/3 complex components, obtained using integration of MAC-tag data compares well with Arp2/3 complex structure. Atomic structure of bovine Arp2/3 complex (PDB code: 4JD2) was used and the Arp2/3 components color-coded (Key: ARPC1B=violet, ARP2=black, ARPC2=blue, ARP3=brown, ARPC3=green, ARPC4=yellow and ARPC5=orange).
