# Supplementary material for: An AP-MS- and BioID-compatible MAC-tag enables comprehensive mapping of protein interactions and subcellular localizations
Source: Nat Commun. 2018 Mar 22;9:1188. doi: 10.1038/s41467-018-03523-2 (PMC5864832; doi:10.1038/s41467-018-03523-2)
Supplement: Supplementary file 3 — Description of Additional Supplementary Files(PDF 165 kb) [file 41467_2018_3523_MOESM3_ESM.pdf]

Description of Additional Supplementary Files

File Name: Supplementary Data 1
Description: Bait proteins information and high-confidence interactions.

File Name: Supplementary Data 2
Description: Matrix for generating heatmap.

File Name: Supplementary Data 3
Description: Application of MS-microscopy on published BioID data.
